# Extensive urine production in euryhaline red stingray for adaptation to hypoosmotic environments

**DOI:** 10.1016/j.isci.2025.113274

**Published:** 2025-08-20

**Authors:** Naotaka Aburatani, Wataru Takagi, Marty Kwok-Shing Wong, Nobuhiro Ogawa, Shigehiro Kuraku, Mana Sato, Kazuhiro Saito, Waichiro Godo, Tatsuya Sakamoto, Susumu Hyodo

**Affiliations:** 1 Atmosphere and Ocean Research Institute, The University of Tokyo, Tokyo, Japan; 2 Department of Genomics and Evolutionary Biology, National Institute of Genetics, Mishima, Japan; 3 Department of Genetics, Sokendai (Graduate University for Advanced Studies), Hayama, Japan; 4 Ushimado Marine Institute, Faculty of Science, Okayama University, Okayama, Japan

**Keywords:** Zoology, Biochemistry, Animal Physiology

## Abstract

Maintaining water balance is a prerequisite for all organisms. Euryhaline elasmobranchs face the severest water-influx potential in fresh water (FW), as they retain high concentrations of urea even in hypotonic environments. To elucidate how they overcome this osmotic challenge, we assessed urine output in conscious euryhaline red stingrays (*Hemitrygon akajei*). Following acclimation to 5% diluted seawater, the stingrays increased urinary output by 87-fold—the greatest change observed in vertebrates—partly due to 6.8-fold increase in glomerular filtration rate (GFR). In the nephron, expressions of *Aquaporin-1* (*Aqp1)*, *Aqp3*, and *Aqp15* were strongly downregulated in FW, indicating that tubular diuresis bridges the gap between GFR and final urine volume. Meanwhile, FW-acclimation upregulated *Aqp1* and *Aqp4* in the distinct bundle structure, which promotes urea reabsorption. Euryhaline elasmobranchs resolve the huge osmotic challenge of FW by excreting massive amounts of water and retaining osmolytes including urea through coordinated regulation of GFR and *Aqp* expressions.

## Highlights

• Our new device enables continuous urine sampling from a conscious red stingray
• Red stingray can increase the urine volume by 87-fold in low salinity conditions
• Urine output increases via enhanced glomerular filtration and reduced water reabsorption
• Segment-specific aquaporin regulation contributes to diuresis and osmolyte retention

## Graphical abstract

**Figure:**
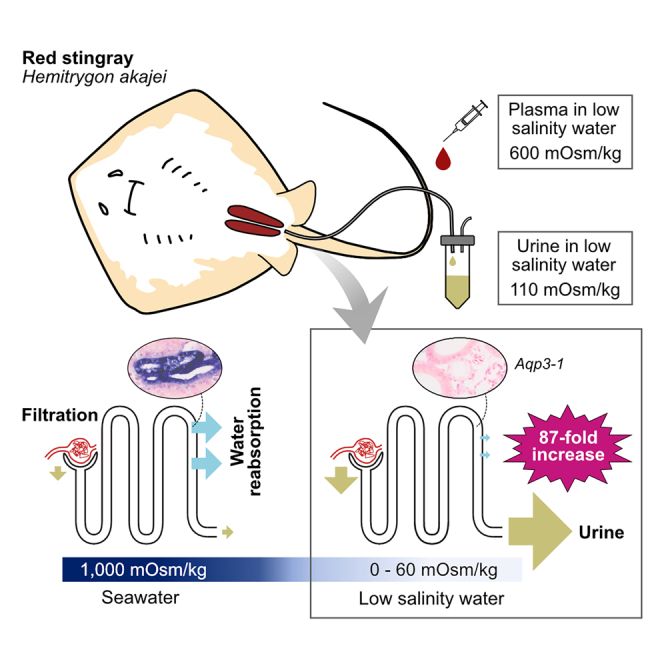

## Introduction

Water homeostasis is fundamental to the survival of all living organisms. However, maintaining it becomes challenging when substantial osmotic gradients arise between body fluids and the surrounding environment. One prominent and unique example is FW-adapted euryhaline elasmobranchs (sharks and rays), which possess the ability to utilize both fresh water (FW) and seawater (SW) in their life cycle.^1^ In the FW environment, these species face the steepest osmotic gradients among FW animals thus far examined.^2^

Most elasmobranchs are marine species, and their body fluid composition is characterized by high concentrations of NaCl and organic osmolytes such as urea.^3^ This composition makes the body fluids iso-osmotic or slightly hyper-osmotic to the high-salinity SW environment, effectively preventing dehydration stress.^4^ Euryhaline elasmobranchs conduct urea-based osmoregulation in SW, similar to the stenohaline marine counterpart. Notably, they maintain high urea concentrations in their body fluid even in the FW environments, while reducing their plasma osmolality down to around 600 mOsm/kg.^5^^,^^6^ This osmoregulatory pattern is clearly distinct from other FW vertebrates, and the resulting marked osmotic gradient between body fluid and ambient water (Δ_osmolality_; about 600 mOsm/kg) causes massive water influx into the body. However, the mechanisms to overcome such an extraordinary osmotic disturbance are not characterized yet.

Compared with elasmobranchs, there are many euryhaline species of teleosts, and their osmoregulatory mechanisms have been extensively documented.^7^^,^^8^^,^^9^ Most teleosts maintain body fluid osmolality ranging from 250 to 400 mOsm/kg, and the osmolality is well maintained within the narrow range.^10^ Therefore, FW teleosts, including euryhaline species, experience osmotic gradients half that of euryhaline elasmobranchs in FW (Δ_osmolality_; often referred to as about 300 mOsm/kg^11^). Following acclimation to an FW environment, euryhaline teleosts, such as Japanese eel (*Anguilla japonica*), winter flounder (*Pseudopleuronectes americanus*) and Atlantic salmon (*Salmo salar*) vigorously increase the urine volume (urine flow rate; UFR) 4.9–6.3 times more than those in SW to eliminate excess water, and this increase is mostly achieved by raising the glomerular filtration rate (GFR).^12^^,^^13^^,^^14^ Then how do the FW-adapted euryhaline elasmobranchs overcome an even greater osmotic gradient compared with teleosts? Janech et al.^15^ demonstrated that euryhaline Atlantic stingray (*Hypanus sabinus*) increases UFR and GFR in 50% diluted SW by 9.0-fold and 3.3-fold, respectively. The observed difference between UFR and GRF suggests that there are particular renal mechanisms to increase UFR, apart from the increase in GFR. However, the study conducted by Janech et al.^15^ had three major limitations: (1) the osmotic gradient between the environment (50% SW, 462 mOsm/kg) and body fluid (Δ_osmolality_; 269 mOsm/kg) was equal to or less than that in FW teleosts, (2) the stingrays were anesthetized to collect urine, a condition known to alter renal function including UFR and GFR,^16^^,^^17^ and (3) they did not examine the underlying molecular mechanisms in the context of the extraordinarily complicated morphology of elasmobranch kidney mentioned further.

The nephron, as the functional unit of the vertebrate kidney, exhibits a unique and complex structure in marine and euryhaline elasmobranchs.^18^^,^^19^ A key morphological feature of elasmobranch kidney is the presence of a cellular peritubular sheath that wraps multiple tubular segments originating from a single nephron. This sheath defines distinct bundle and sinus zones in the kidney, across which the nephron repeatedly traverses to form four loops. Furthermore, the nephron is divided into as many as 16 segments that express individualized functions, making it the most complicated among vertebrates including teleosts and mammals.^20^^,^^21^^,^^22^ As for euryhaline species, for instance, the upregulation of sodium chloride-transporting machineries was recently found in a particular distal segment in FW-acclimated red stingray (*Hemitrygon akajei*)^23^ and bull shark (*Carcharhinus leucas*).^24^ We therefore expected that regulation of water and solute transport in specialized segments enables euryhaline elasmobranchs to overcome the massive osmotic disturbance in FW. One plausible mechanism is tubular water reabsorption, well-known in the mammalian kidney, where water loading increases UFR by suppressing water reabsorption via aquaporin-2 (AQP2; major renal water channel to reabsorb filtered water among 13 known AQPs in mammals^25^) at the inner medullary collecting duct (IMCD).^26^^,^^27^^,^^28^ To explore the renal mechanisms enabling euryhalinity in elasmobranchs, comprehensive physiological, and molecular investigation is required.

We therefore developed a method to assess the GFR and UFR in conscious stingrays, allowing us to uncover the physiological adaptations of euryhaline elasmobranchs during transitions from full-strength SW to diluted (5%) SW, and vice versa. Moreover, through a genome-wide analysis followed by mRNA expression analysis, we examined dynamic fluctuations in the AQP repertoire of the stingray kidney to explore the contribution of tubular diuresis. These approaches allowed us to overcome the limitations of previous studies and highlight the significance of renal function in euryhaline elasmobranchs.

## Results

### Changes in plasma and urine compositions following the acclimation to low environmental salinity

Table 1 shows the compositions of plasma and urine in SW and following acclimation to 5% SW and return to SW (RSW). In SW, plasma contained high levels of NaCl and urea, resulting in high osmolality that was nearly isotonic to the surrounding SW (plasma, 1073.6 ± 7.0 mOsm/kg; environmental water, 1077.4 ± 3.3 mOsm/kg). These plasma parameters were significantly decreased following the acclimation to 5% SW, however, considerable levels of NaCl and urea remained in the plasma, forming a steep osmotic gradient between the plasma (603.6 ± 16.9 mOsm/kg) and environment (5% SW; 55.9 ± 4.3 mOsm/kg). After RSW-acclimation, the plasma NaCl levels were restored to the initial SW levels, and plasma osmolality rose to 969.4 ± 12.1 mOsm/kg, which was slightly lower than the environmental osmolality (RSW; 1061.1 ± 7.9 mOsm/kg).

**Table 1:** The compositions of plasma and urine from SW control, 5% SW- and RSW-acclimated red stingray

	*n*	Osmolality (mOsm/kg)	Na^+^ (mM)	Cl^−^ (mM)	Urea (mM)
**Plasma**					

SW	5	1073.6 ± 7.0^a^	334.2 ± 6.0^a^	283.8 ± 1.1^a^	419.4 ± 6.4^a^
5% SW	8	603.6 ± 16.9^b^	200.0 ± 5.9^b^	167.5 ± 6.8^b^	212.8 ± 9.2^b^
RSW	6	969.4 ± 12.1^c^	321.2 ± 7.1^a^	289.3 ± 3.8^a^	321.3 ± 7.0^c^

**Urine**					

SW	5	1046.5 ± 13.0^a^	341.5 ± 38.5^a^	187.6 ± 57.0^a^	81.9 ± 14.2^a^
5% SW	8	113.9 ± 16.3^b^	19.7 ± 4.8^b^	13.2 ± 3.7^b^	59.2 ± 11.3^a^
RSW	6	865.3 ± 67.2^c^	240.5 ± 41.2^a^	114.4 ± 25.8^b^	75.0 ± 13.0^a^

**Environmental water**					

SW	5	1077.4 ± 3.3^a^	563.1 ± 7.6^a^	563.5 ± 2.7^a^	<1.0
5% SW	8	55.9 ± 4.3^b^	26.2 ± 2.7^b^	25.3 ± 2.1^b^	<1.0
RSW	6	1061.1 ± 7.9^a^	571.6 ± 9.7a	565.5 ± 5.5^a^	<1.0

Different letters indicate statistically significant differences.

In the SW group, the osmolality and sodium ion levels of urine were almost identical to those of plasma. Meanwhile, the concentrations of chloride ion and urea in urine were lower than those in plasma. All urinary solute concentrations except for urea were significantly decreased following the acclimation to 5% SW, resulting in a lower osmolality of urine (113.9 ± 16.3 mOsm/kg) compared to that of the plasma (603.6 ± 16.9 mOsm/kg), and to that of urine in SW individuals (1046.5 ± 13.0 mOsm/kg). Urine concentrations of sodium and chloride ions were also elevated by the RSW transfer, although the urine chloride ion levels in the RSW group were significantly lower than those in SW group. Consequently, the difference between the osmolalities of urine and plasma in 5% SW-acclimated stingray was reduced in the RSW condition (urine, 865.3 ± 67.2 mOsm/kg; plasma, 969.4 ± 12.1 mOsm/kg).

### Dynamics of UFR and GFR in different salinities

**Figure 1:**
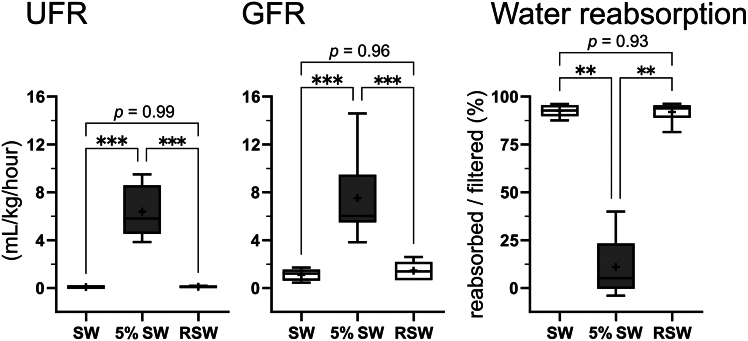
The urine flow rates (UFR), glomerular filtration rates (GFR) and the percentages of water reabsorption in the red stingrays Values were expressed as box-scatter plots (Box, first and third quartiles; scatters, minimum and maximum values; plus signs, average; bar inside box, median). Statistically-significant difference among groups is shown with asterisks (∗∗∗*p* < 0.001, ∗∗*p* < 0.01, *n* = 5, 8, and 6 for SW, 5% SW, and RSW groups, respectively).

**Figure 2:**
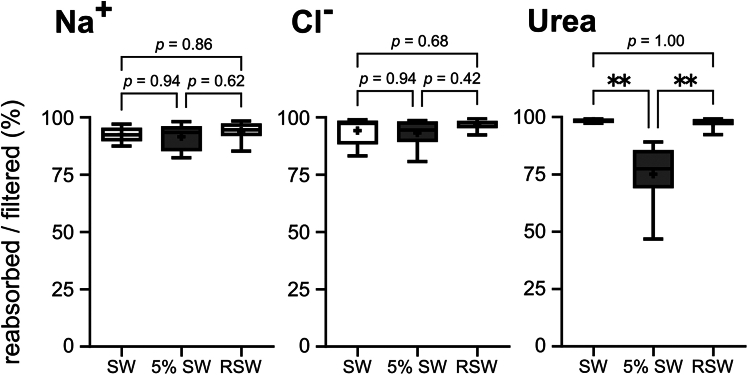
The percentage of reabsorbed solutes per filtered in the kidney of red stingray Values were expressed as box-scatter plots (Box, first and third quartiles; scatters, minimum and maximum values; plus signs, average; bar inside box, median). Statistically-significant difference among each group is shown with asterisks (∗∗*p* < 0.01, *n* = 5, 8, and 6 for SW, 5% SW and RSW groups, respectively).

In our preliminary experiment, injected inulin was stably detected from 6 h to at least up to 48 h after the injection in SW individuals (open markers in Figure S1). In 5% SW-acclimated individuals, plasma inulin levels sharply increased at 1 h after the injection, and gradually decreased from 12 h to 48 h (filled markers in Figure S1). These results indicate that no significant change in the plasma inulin level is anticipated during 24–48 h after the injection in both SW- and 5% SW, and thus we collected urine between 24 h and 48 h after the inulin injection for 2–5 h in SW and RSW individuals and for 0.5 h in 5% SW individuals. This resting time after the surgery was also advantageous to alleviate possible disturbance of renal functions by the anesthesia and surgical stress. The calculated UFR, GFR, and the percentages of water reabsorption are presented in Figure 1. The UFR was significantly increased by 87 times following the transfer from SW to 5% SW, and decreased to the initial SW levels after RSW acclimation (SW, 0.07 ± 0.01 mL/kg/h; 5% SW, 6.39 ± 0.75 mL/kg/h; RSW, 0.11 ± 0.03 mL/kg/h). The GFR was also significantly increased by 6.8 times in 5% SW and then returned to a low level in RSW (SW, 1.11 ± 0.22 mL/kg/h; 5% SW, 7.52 ± 1.21 mL/kg/h; RSW, 1.46 ± 0.35 mL/kg/h). As a result, the percentages of water reabsorption were significantly decreased in 5% SW, compared to those in SW and RSW groups, indicating that tubular water reabsorption was suppressed in 5% SW environment (SW, 92.7 ± 1.5%; 5% SW, 11.0 ± 5.4%; RSW, 92.0 ± 2.2%). We further calculated solute reabsorption rate in the kidney. The solute reabsorption rates of all examined ions and urea were significantly higher in 5% SW compared to SW or RSW, and no significant difference was observed between SW and RSW (Table 2). As a result, percentages of Na^+^ and Cl^−^ reabsorption were consistently high regardless of the external salinities (Figure 2; Na^+^: 92.5 ± 1.5%, 91.6 ± 2.0%, 94.0 ± 1.9%; Cl^−^: 94.2 ± 2.9%, 93.2 ± 2.1%, 96.9 ± 1.0% for SW, 5% SW, and RSW, respectively). Although the percentage of urea reabsorption was significantly decreased in 5% SW-acclimated individuals, more than 75% of filtered urea was reabsorbed in 5% SW (Figure 2; 98.5 ± 0.4%, 75.2 ± 4.9%, 97.8 ± 1.1% for SW, 5% SW, and RSW, respectively).

**Table 2:** Solute reabsorption rates (μmol/kg/h) in each condition

	*n*	Na^+^	Cl^−^	Urea
SW	5	349.3 ± 79.1^a^	298.7 ± 64.6^a^	455.5 ± 92.9^a^
5% SW	8	1401.5 ± 250.3^b^	1209.5 ± 239.1^b^	1285.5 ± 321.7^b^
RSW	6	450.4 ± 113.8^a^	411.8 ± 101.2^a^	464.7 ± 116.6^a^

Different letters indicate statistically significant differences.

### Expression of water and solute transporting proteins with identification of the red stingray aquaporin genes

**Figure 3:**
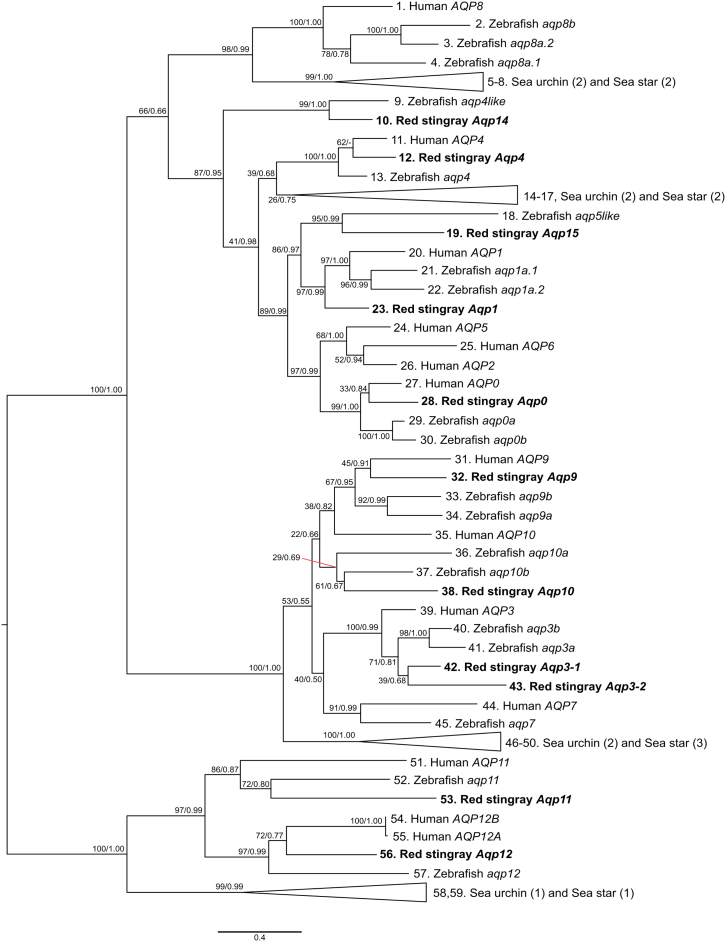
Molecular phylogeny of the red stingray aquaporin genes Their phylogenetic relationships in the entire aquaporin gene family was inferred with maximumlikelihood method with 266 residues in the amino acid sequence alignment (see materials and methods). The support values at nodes are bootstrap probabilities in the ML tree and posterior probabilities in the Bayesian inference in order (see materials and methods). The numbers in the parentheses show the multiplicity of the sequences used for the outgroup species.

We next focused on expression of aquaporins to elucidate molecular mechanisms of extensive diuresis. Prior to the renal profiling of *Aqp* expressions, we examined red stingray *Aqp* gene repertoires based on its whole genome sequence data (NCBI Assembly ID: JBLLJJ000000000). Our phylogeny inference of the entire *Aqp* gene family showed explicit groupings of the red stingray *Aqp* genes into existing subfamilies (*Aqp0*, -*1*, -*3*, -*4*, -*9*, -*10*, *-11*, *-12*, and -*14*) (Figure 3). The phylogenetic tree indicated that the Elasmobranchii including the red stingray experienced *Aqp3* gene duplication (named *Aqp3-1* and -*3-2*), independently of the gene duplication that gave rise to the two *aqp3* genes in teleosts (e.g., zebrafish [*Danio rerio*] *aqp3a* and -*3b*^29^) (Figure S2).

**Figure 4:**
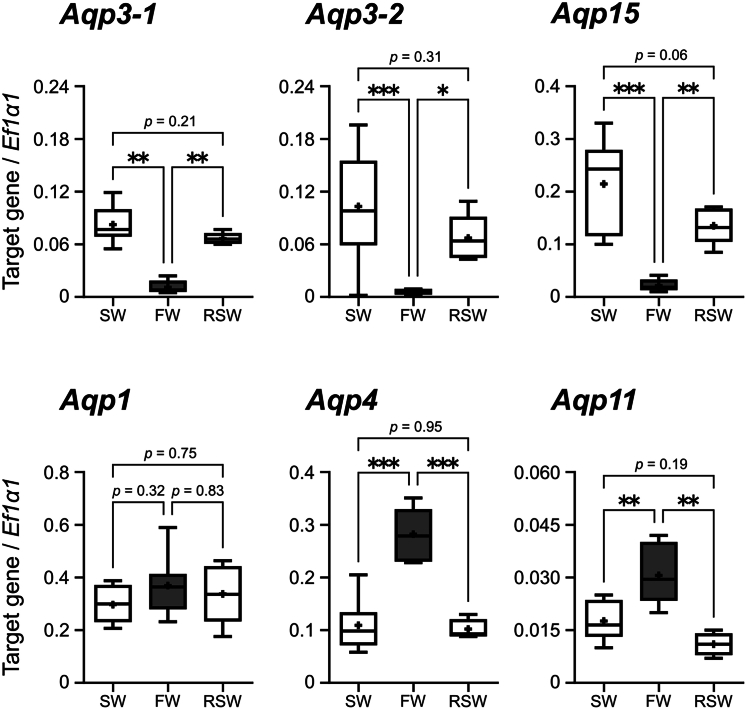
Expression of *Aqp*s mRNA in the stingray kidney quantified by RT-qPCR The expression levels were normalized against elongation factor 1α1 (*Ef1α1*). Values were expressed as box-scatter plots (Box, first and third quartiles; scatters, minimum and maximum values; plus signs, average; bar inside box, median). Asterisks indicate statistically significant difference (∗*p* < 0.05, ∗∗*p* < 0.01, ∗∗∗*p* < 0.001, *n* = 8, 8, and 5 for SW, FW, and RSW groups, respectively. Note that the expression levels of *Aqp11* were much lower than other *Aqp*s.

Tissue distribution analysis indicated that *Aqp9* and -*10* were not expressed in kidney but abundantly expressed in extrarenal organs such as liver, stomach and intestine (Figure S3). Renal transcriptome data obtained in our previous investigation^23^ allowed us to set a cutoff criterion (*Aqp*s with transcripts per million [TPM] > 5 in any of six samples), and we focused on *Aqp1*, -*3-1*, -*3-2*, -*4*, -*11*, and -*15* (Table S1). Among these *Aqps*, the TPM values of *Aqp3-1*, -*3-2*, and -*15* were lower in FW than SW by one-sixth or less. On the other hand, the TPM values of *Aqp4* and *11* were higher in FW than SW (4.2 and 3.0 times for *Aqp4* and -*11*, respectively), while those of *Aqp1* varied among individuals. These expression patterns were further confirmed by qPCR analysis (Figure 4). Furthermore, the expression levels of *Aqp*s in RSW-acclimated stingrays were nearly identical to those in SW controls. No difference was observed in the expression levels of *Aqp1* among SW, FW, and RSW. Of note, the expression levels of *Aqp11* were one order of magnitude lower than other *Aqp*s (less than 0.042 copies/*Ef1α1*).

**Figure 5:**
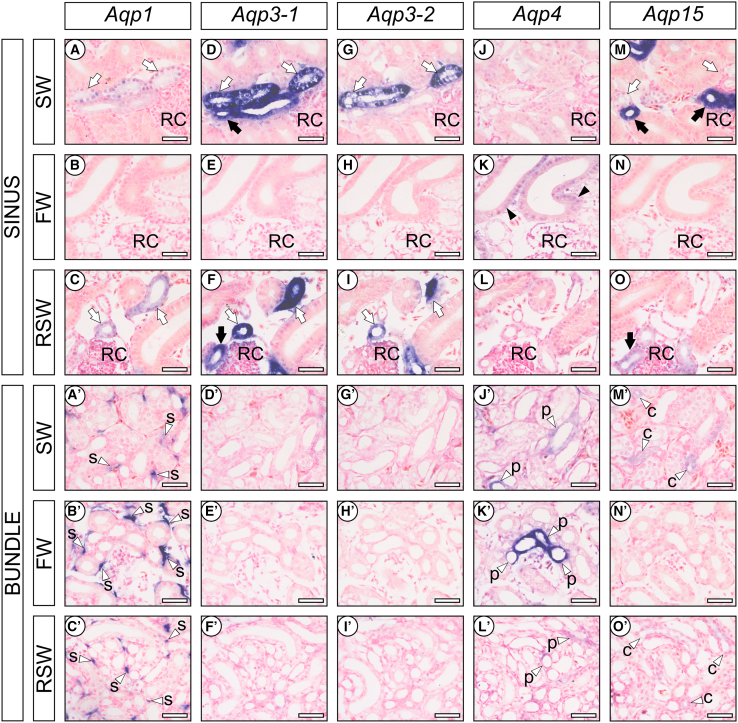
*In situ* hybridization analysis on *Aqp*s in the red stingray nephron The respective mRNA signals in sinus zone (A–O) and bundle zone (A′ –O′) of *Aqp1* (A–C and A′–C′), *3-1* (D–F and D′–F′), *3-2* (G–I and G′–I′), *4* (J–L and J′–L′), and *15* (M–O and M′–O′) are shown. Filled arrowheads indicate the PII (K). Open and filled arrows indicate the anterior and posterior LDT, respectively (A–I and M–O). RC, renal corpuscle. Open arrowheads indicate the peritubular sheath (labeled with “s”), PI (labeled with “p”), and CT (labeled with “c”) in the bundle zone (A′-B′ and J′-O′). Scale bars, 50 μm. Note that (1) in the sinus zone of FW-acclimated stingray, the mRNA signals of *Aqp1*, *3-1*, *3-2*, and *15* were almost undetectable, while (2) in the bundle zone, intense mRNA signals of *Aqp1* and *4* were observed in the peritubular sheath and PI of FW-acclimated stingrays, respectively, compared with SW control and RSW-acclimated individuals.

The kidney of marine and euryhaline elasmobranchs is composed of two anatomically distinct zones, the sinus and bundle zones, and each nephron traverses repeatedly between the two zones to form a “four-loop nephron”.^18^^,^^19^ In the sinus zone, intense mRNA signals of *Aqp1,* -*3-1*, -*3-2*, and -*15* were detected in the late distal tubule (LDT) of both SW and RSW groups of stingrays. The anterior LDT expressed *Aqp1*, -*3-1*, and -*3-2* (open arrows in Figures 5A–5D and 5G), while the posterior LDT expressed *Aqp3-1* and -*15* (filled arrows in Figures 5D and 5M). In the sinus zone of the FW-acclimated stingray, no intense signals of *Aqp1*, -*3-1*, -*3-2*, and -*15* were detected, while expression of *Aqp4* was only observed in the largest diameter tubule of proximal II (PII, filled arrowheads in Figure 5K).

In the bundle zone, mRNA signals of *Aqp1*, -*4* and -*15* were detected. In all groups, the mRNA signals of *Aqp1* were localized in renal interstitial cells surrounding the nephron segments of bundle zone (open arrowheads labeled “s” in Figures 5A′, 5B′ and 5C′), but not in tubular cells. This suggests that the *Aqp1* is expressed in the peritubular sheath. On the other hand, mRNA signals of *Aqp4* were localized in PAS-positive segments of the bundle zone (open arrowheads labeled “p” in Figures 5J′, 5K′ and 5L′; open arrows in Figure S4) with absence of Na^+^-K^+^-Cl^-^ cotransporter 2 (*Nkcc2*) and Urea transporter (*Ut*) mRNA signals, which are marker genes for early distal tubule (EDT) and collecting tubule (CT), respectively (Figure S4). By elimination, the results indicate that *Aqp4* is expressed in the proximal tubule I (PI). The signal intensities of both *Aqp1* and -*4* in the bundle zone were stronger in FW individuals than in SW and RSW groups. Weak signals of *Aqp15* were detected in CT of SW and RSW groups with co-localization of *Ut* mRNA, a marker gene for CT (open arrowheads labeled with “c” in Figures 5M′, 5O′, and S5). In the FW-acclimated individuals, however, no intense signal of *Aqp15* was observed (Figure 5N′). Although *in situ* hybridization for *Aqp11* was performed, no signal was detected probably due to the low expression levels (Figure 4).

We also confirmed our previous observation that *Nkcc2* and Na^+^/K^+^ ATPase alpha 1 subunit (*Nkaα1*) mRNA levels were increased in the EDT following the transfer of stingray from SW to FW (Figures S6A, S6B, S7A′, S7B′, S7D′, and S7E′). In addition, the present investigation revealed that the elevated expressions in *Nkcc2* and *Nkaα1* mRNAs were decreased to the initial SW levels following the transfer to RSW (Figures S6A, S6B, S7C′, and S7F′). On the other hand, the *Ut* mRNA was expressed at similar levels in all experimental groups (Figure S6C). However, *in situ* hybridization revealed that *Ut* mRNA expression was differently regulated among the separate nephron segments. In FW-acclimated individuals, the signal intensity of *Ut* mRNA was stronger in the CT while weaker in the LDT than SW controls and RSW-acclimated individuals (Figures S7G′–S7I′ and S7G–S7I).

### Fine structure of peritubular sheath and the expression of *Aqp1*

**Figure 6:**
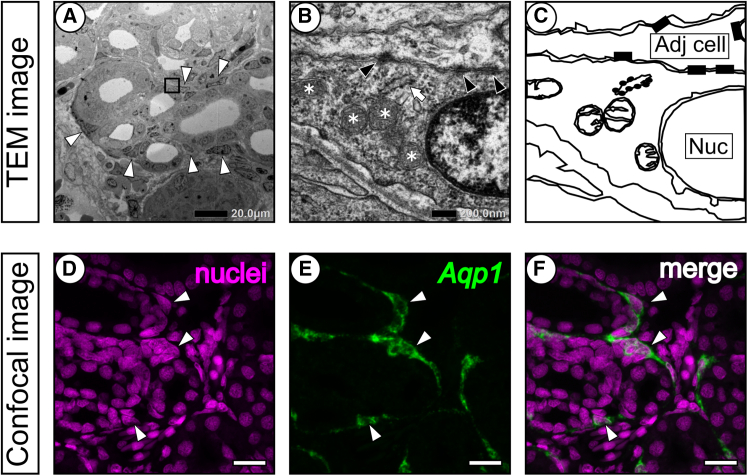
Identification of cells expressing *Aqp1* in the kidney (A–C) Fine structure of the peritubular sheath cells was observed by TEM. The peritubular sheath consisted of several cells (A; open arrowheads, nuclei) surrounding the nephric tubules of the bundle. Approximate position of magnified view in (B) is shown as a square window in panel (A). Asterisks, filled arrowheads, and an open arrow represent mitochondria, tight junctions, and an electron dense organelle, respectively. Schematic drawing of (B) is shown in (C; Nuc; nuclei, Adj cell; Adjacent cell of peritubular sheath). (D–F) Nuclear localization of peritubular sheath and expression of *Aqp1* mRNA were visualized by a confocal laser microscope. Fluorescent mRNA signals of *Aqp1* were detected in peritubular sheath cells (open arrowheads). Scale bars, 20 μm (A, D, E, and F) or 200 nm (B).

To further resolve the localization of *Aqp1* mRNA, fine structure was observed by transmission electron microscopy (TEM), and fluorescence in situ hybridization (FISH) signal was observed by confocal laser microscope. In low magnification view of TEM, peritubular sheath was observed as it surrounds the renal tubules in the bundle zone (Figure 6A). Each cross section of the sheath contains five to seven cells deduced by the number of nuclei (open arrowheads in Figure 6A). Under a higher magnification view, these cells were thinly projected and overlapped with the adjacent cells (Figure 6C) with multiple tight junctions at the cell boundary (filled arrowheads in Figure 6B). Mitochondria (asterisks in Figure 6B) and an electron-microscopically dense other organelle (presumably rough endoplasmic reticulum, open arrow in Figure 6B) were near the nuclei in peritubular sheath cells. These organelles were not observed in peripheral portions of the adjacent cells (Figures 6B and 6C). Using double staining of the nuclei and *Aqp1* mRNA, expression of *Aqp1* was detected in the cytoplasm of renal interstitial cells that enclosed the renal tubules in the bundle zone (Figures 6D–6F). These results indicate that the extra-tubular NBT/BCIP signals of *Aqp1* mRNA in the bundle zone (Figures 5A′–5C′) is on the peritubular sheath cells with a perinuclear mRNA localization.

## Discussion

Acquisition of euryhalinity has played a pivotal role in habitat expansion of aquatic life forms. Class Elasmobranchii is a mostly marine-limited group, and euryhaline species are few and exceptional.^6^^,^^30^ Retaining high internal concentrations of urea in FW makes their plasma osmolality exceptionally high among freshwater animals.^2^ However, it has been a long-lasting enigma as to how they deal with the resulting massive water influx and how renal function, particularly including urine volume regulation, is dynamically adjusted according to change in environmental salinity. In the present study, we demonstrated, for the first time, that acclimation of red stingray to 5% SW induces an 87-fold increase in UFR, which is greater than that seen in any other vertebrate thus far examined.^26^ Especially, it provides a striking contrast to the relatively modest capacity for water excretion in euryhaline teleosts for which UFR values are just 4.9–6.3 times higher in FW than in SW.^12^^,^^13^^,^^14^ Although such a massive increase in UFR could potentially lead to significant solute loss, we found that plasma solute concentrations were maintained above a certain level even under hypoosmotic conditions likely due to enhanced renal solute reabsorption. Herein, we propose plausible mechanisms enabling water excretion and solute retention simultaneously in the elaborate four-loop nephron.

### Evaluation of GFR and UFR in red stingray under varied salinity conditions

By developing a method to evaluate renal functions of red stingray under unanesthetized conditions, we revealed dynamic changes in GFR and UFR during sequential transfers to low-salinity (5% SW) and return to full-strength SW. The UFR values (0.07 ± 0.01 mL/kg/h) of red stingray in SW are similar to those of conscious lesser-spotted dogfish (*Scyliorhinus canicula*; <0.1 mL/kg/h^31^) and little skate (*Leucoraja erinacea*; 0.19 mL/kg/h^32^) in SW. Meanwhile, the GFR (1.11 ± 0.01 mL/kg/h) and UFR of red stingray were lower than the GFR (3.8 ± 0.8 mL/kg/h) and UFR (0.9 ± 0.2 mL/kg/h) in anesthetized Atlantic stingray in SW. Therefore, it appears that our method successfully evaluated renal functions in red stingray, and the previous study by Janech et al.^15^ might have overestimated both GFR and UFR in SW, presumably due to anesthesia during urine collection.

In stingrays acclimated to 5% SW, the 87-fold increase in UFR could not be achieved solely by the 6.8-fold increase in GFR. We observed a simultaneous large decrease in the tubular water reabsorption, indicating that the excretion of such a massive amount of dilute urine is achieved by regulations of both GFR and tubular water reabsorption. Our data showed that 93.7% of filtrated water was reabsorbed during urine production in SW (GFR, 1.11 mL/kg/h; UFR, 0.07 mL/kg/h), while the reabsorption fraction decreased to 15% in 5% SW (GFR, 7.52 mL/kg/h; UFR, 6.39 mL/kg/h). This regulation pattern is distinct from teleosts where change in GFR is mostly responsible for the change in UFR,^28^ and is also distinct from that in water-loaded mammals where decreasing tubular water reabsorption contributes primarily to the increase in UFR (up to 40-folds increase in UFR without changing GFR^26^^,^^33^). Furthermore, since all of the altered renal parameters in stingrays acclimated to 5% SW were restored to initial values by transfer back to SW, the stingray kidney demonstrated its adaptability to a wide range of salinity changes.

### Downregulation of *aqp*s in the sinus zone: A crucial mechanism to increase UFR

Through multifaceted approaches including genome search, we identified the same gene repertoires as those of the zebra shark (*Stegostoma tigrinum*),^34^ except that our search did not detect duplicated *Aqp10* orthologs. Among the identified stingray genes, five *Aqp* genes highly expressed in the stingray kidney. The remarkable finding was that, among the five *Aqp* genes, three genes (*Aqp3-1*, -*3-2*, and -*15*) were downregulated in low-salinity environment, whereas two genes (*Aqp4* and -*1*) were upregulated in a segment-specific manner. We presume that the oppositely-directed regulation of *Aqp*s is necessary to achieve two separate demands in FW, namely, water excretion and solute retention. Indeed, the downregulated and upregulated *Aqp* genes showed distinct localization in the kidney.

**Figure 7:**
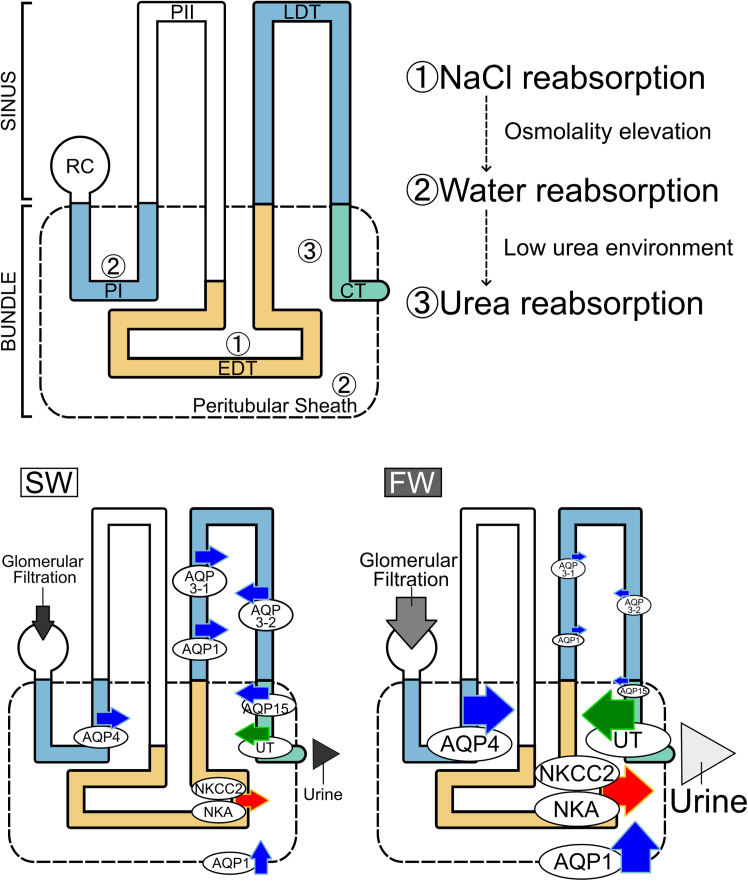
Schematic summary for the urea reabsorption and water handling dynamics in the kidneys of red stingray Gray arrows and triangles indicate glomerular filtration and urine flow, respectively. The thickness of gray colors reflects the strength of osmolality. Arrows in blue, red and green represent reabsorption of water, NaCl and urea via aquaporins and transporters, respectively. In FW, glomerular filtration rate is increased. Simultaneously, AQP1, 3-1, 3-2, and 15 were downregulated through the LDT to CT to suppress water reabsorption, while AQP4 in the PIb and AQP1 in peritubular sheath were upregulated to supply water into micro-environment inside bundle zone for low-urea environmentalization and urea reabsorption. These coordinated regulations contribute to the production of pronounced amount of dilute urine in FW. RC, renal corpuscle.

Expressions of *Aqp1*, -*3-1*, -*3-2*, and -*15* were intensely detected in the LDT of sinus zone, the 4th loop of the nephron, in SW and RSW. Signal intensities in the LDT were markedly decreased following the transfer to FW. These results suggest that AQP1, -3-1, -3-2, and −15 are involved in the tubular water reabsorption, and that the downregulation of *Aqp1,* -*3-1,* -*3-2*, and -*15* in the LDT induces water excretion in the FW environment. In the elasmobranch four-loop nephron, the EDT is a diluting segment, in which NaCl is actively reabsorbed via NKA and NKCC2.^24^^,^^35^^,^^36^ As the EDT (3rd loop) is located in advance of the LDT (4th loop) (see Figure 7), the desalinated filtrate must naturally flow into the LDT. Therefore, the LDT is the most appropriate segment for tubular water reabsorption in the SW environment, and the suppressed water reabsorption by the reduced *Aqp* expressions causes large urine output following FW acclimation. This is analogous to the tubular diuretic regulation by AQP2 in the IMCD of mammalian kidney, where downregulation of *Aqp2* and internalization of AQP2 play a pivotal role to increase UFR in a water-loaded condition.^27^ On the other hand, the EDT in the elasmobranch nephron with its expression of NKA and NKCC2 is functionally similar to the thick ascending limb of loop of Henle in the mammalian nephron.^36^^,^^37^

A few studies have demonstrated that the renal mRNA expressions of *aqps* are downregulated in FW-acclimated euryhaline teleosts.^38^^,^^39^^,^^40^ However, these *aqp*s were still intensely detected also in FW condition, unlike the faint staining of stingray *Aqp3-1*, -*3-2*, and -*15* in FW individuals. Taken together with the minor decrease in the tubular water reabsorption in FW-acclimated euryhaline teleosts, the contribution of AQPs to controlling UFRs is suggested to be relatively insignificant in teleost fishes. In contrast, to satisfy the higher demand for water excretion in FW than that in teleosts, euryhaline elasmobranchs have likely evolved dynamic regulation of AQPs at the LDT, in addition to increasing GFR, to efficiently increase UFR in FW.

### Upregulation of *aqp*s in the bundle zone: A possible contribution to the urea reabsorption process

In contrast to downregulation of *Aqp*s in the LDT, significant upregulation of *Aqp1* and -*4* mRNAs were found in the bundle zone of the stingray kidney following the FW acclimation. These findings suggest that AQP1 and -4 within the bundle zone have functions distinct from the control of UFR.

In marine and euryhaline elasmobranchs, the bundle zone contains multiple segments (PI, intermediate tubule, EDT and CT) originated from a single nephron, which are wrapped by the impermeable peritubular sheath (see Figures 6A–6C).^41^ This characteristic structure is considered to be important for urea reabsorption in elasmobranch nephron,^42^ and we proposed a model for the urea reabsorption in the bundle zone based on molecular mapping data.^20^^,^^23^ By this model, three steps are thought to be essential for successful urea reabsorption: (1) active reabsorption of NaCl, (2) passive reabsorption of water, and (3) facilitative reabsorption of urea (Figure 7). The first step involves active transport of NaCl from primary urine into the interstitial space within the peritubular sheath. The NKCC2 and NKA expressed in the EDT^24^^,^^36^^,^^43^^,^^44^ are responsible for the transport of NaCl, which results in elevation of interstitial osmolality inside the peritubular sheath. For the second step, the elevated interstitial osmolality leads to water reabsorption from the filtrate to the interstitial microenvironment via AQPs to create a low-urea fluid inside the peritubular sheath. After these two steps, urea is concentrated in the filtrate and tubular water reabsorption in the LDT further increases the luminal urea concentrations. In the final step, urea is reabsorbed through the facilitative UT expressed in the CT, driven by the concentration gradient of urea between the interstitial micro-environment and processed filtrate in the CT.^36^^,^^44^^,^^45^ Based on this model, the third step, namely facilitative urea reabsorption driven by low urea concentration within the bundle, appears to depend on water reabsorption mediated by AQPs in the bundle zone.

In accordance with the proposed transport model, we detected expression of *Aqp1* in the peritubular sheath cells and *Aqp4* in the PI segment in the bundle zone. In the kidney of spiny dogfish (*Squalus acanthias*), immunoreactive AQP4 and -15 were detected in the EDT of the bundle zone and the peritubular sheath.^46^^,^^47^ Even though localization in the renal tubule and type of AQP genes are different between the two studies, AQPs expressed in the tubular bundle and/or peritubular sheath appear to play a role in water uptake into the microenvironment in the bundle zone, and to contribute to the reabsorption of the essential solute, urea, in marine and euryhaline elasmobranchs.

The notion that AQP1 and -4 may contribute to urea reabsorption is supported by the finding of upregulated expression in FW-acclimated individuals. We previously reported upregulations of active NaCl transporters in the EDT in FW-acclimated stingray, which results in more prominent elevation of osmolality inside the bundle zone in FW individuals than in SW individuals.^23^ Consistent with this result, expressions of *Nkcc2, Nkaα1*, *Ut*, and aquaporins (*Aqp1* and -*4*) were upregulated in the bundle zone following the transfer to FW and were restored to the initial SW levels after re-acclimation to SW. With the concurrent increase in AQP1 and -4, elevated intra-sheath osmolality leads to greater inflow of water inside the bundle zone, which eventually facilitates urea reabsorption.

Mechanisms for the oppositely-directed regulation of *Aqp*s between the bundle and sinus zones are quite intriguing. In the mammalian kidneys, vasopressin is a key hormone controlling urine volume and osmolality via activation of transcription and phosphorylation of AQP2.^27^^,^^48^^,^^49^ In this regulatory mechanism, the V2-type receptor (now designated as V2a-type) mediates the antidiuretic action of vasopressin, although cartilaginous fish lack both the V2a-type receptor^50^ and AQP2 (Figure 3). Furthermore, AQP2, -3, and -4 are co-expressed in the IMCD of mammalian nephron where AQP3 and -4 are constitutively located in the basolateral membrane of IMCD cells, while membrane localization of AQP2 in the apical membrane of IMCD is regulated by vasopressin.^51^ Therefore, regulatory mechanism(s) and subcellular localization of AQPs in stingray nephron are an important issue to be clarified in future study. Beside the kidney, extra-renal expression of AQPs is also interesting. In the present study, expression of *Aqp3-1* was extensively downregulated in the kidney, while that in the gill seemed to be upregulated in FW (Figure S3). Similar results have been obtained for branchial *Aqp3* in euryhaline teleosts, such as medaka (*Oryzias latipes*), killifish (*Fundulus heteroclitus*), and milkfish (*Chanos chanos*).^52^^,^^53^^,^^54^ Physiological significances, as well as their regulatory mechanisms, of AQPs in different tissues are thus important subjects of future research.

### Urea retention? Or excretion?

It has been controversial whether urea is excreted or reabsorbed when euryhaline species are acclimated to low-salinities, since plasma urea concentrations were decreased following the transfer to diluted environment in many species including red stingray.^24^^,^^55^^,^^56^ In the present study, we showed that the tubular reabsorption rate of urea was increased 2.5 times in 5% SW, resulting in as high as 75% of filtered urea being reabsorbed from the primary filtrate. Furthermore, expressions of the molecular machinery to reabsorb urea, such as *Nkcc2, Nkaα1*, *Ut*, *Aqp1*, and *Aqp4* in the bundle zone, were all upregulated following transfer to an FW environment. These results reinforced our notion that the kidney of FW-acclimated stingray actively reabsorbs urea as much as possible, rather than excrete it to the environment. Ballantyne and Fraser^57^ pointed out the possibility that FW-acclimated euryhaline elasmobranchs need urea to maintain proper enzymatic activity. Although urea generally alters protein conformation and acts as an enzyme inhibitor,^2^ some proteins of marine elasmobranch are known to require a high concentration of urea to exert proper function.^58^^,^^59^^,^^60^ In addition to the biochemical evidence, our data clearly showed that euryhaline red stingray actively retains a high concentration of urea using the outstanding ability to reabsorb urea in the elaborate nephron, which might be a prerequisite for euryhalinity in elasmobranchs and for survival in low salinity environment.

Plasma osmolality in RSW individuals was not completely restored to the original level measured in SW, mainly due to a lower plasma urea level in the RSW group compared to the SW group. Considering that urea synthesis consumes ATP energy,^2^ the experimental design in which the stingrays were kept starved may have affected the urea accumulation process in RSW, but not the NaCl regulation.

### Conclusion

Herein, we provided evidence that euryhaline red stingray possesses great plasticity in renal functions that enables considerable increases in UFR. This ability is essential to solve the dilemma in FW-acclimated euryhaline elasmobranchs that they must excrete massive amounts of urinary water and simultaneously retain a large fraction of filtered solutes including urea in the elaborate four-loop nephron. To the best of our knowledge, the observed 87-fold increase in UFR is one of the largest changes in reported vertebrate renal function, which highlights the exceptional ability of euryhaline species to adapt to a wide range of salinities from SW to FW despite a high plasma osmolality. Although all cartilaginous fishes conducting urea-based osmoregulation have the four-loop nephron in the kidney, only a limited few species including the red stingray have acquired broad euryhalinity. Furthermore, it is likely that euryhaline species have appeared independently multiple times in the elasmobranch lineage.^57^ The mystery of evolution in euryhaline elasmobranchs will be revealed through the comprehensive study of differences between euryhaline and stenohaline species in the future.

### Limitations of the study

We developed our discussion based on the urea reabsorption model of Hyodo et al.^24^ (shown here in Figure 7), but it is important to note that this model primarily relies on molecular mapping data obtained through *in situ* hybridization. In addition, some aquaporins are known to facilitate transport of organic osmolytes such as urea.^33^ Therefore, determining the direction of solute and water transports,^61^ as well as the transport properties of aquaporins using oocyte expression assays,^62^ would further strengthen our model.

## Resource availability

### Lead contact

Further information and requests for resources and reagents should be directed to and will be fulfilled by the lead contact, Wataru Takagi (watarutakagi@aori.u-tokyo.ac.jp).

### Material availability

Plasmids used for generating ISH probes can be requested from the lead contact.

### Data and code availability

All raw sequencing data generated in this study have been deposited at NCBI Bio Project database and are publicly available as of the date of publication. Accession number is listed in the key resources table.

## Acknowledgments

We thank Dr. C. A. Loretz of State University of New York at Buffalo for critical comments on the manuscript, Dr. Shinji Kanda for supporting the fluorescence observation, and Dr. Sadaaki Kayama for the animal collection. This study was supported by Grants-in-Aid for Scientific Research from the 10.13039/501100001691Japan society for the Promotion of Science to S.H. (JSPS KAKENHI 17H03868 and 19K22414), and a Grant-in-Aid for 10.13039/501100001691JSPS Fellows to N.A. (21J20882).

## Author contributions

N.A., W.T., and S.H. conceptualized and designed the study. N.A., W.T., M.K.-S.W., N.O., S.K., M.S., C.T., and M.K.-S.W. performed the experiments and collected data. N.A., W.T., K.S., W.G., and T.S. kept the animals and contributed to sample collection. N.A. wrote the first draft of the manuscript, and W.T., M.K.-S.W., S.K., and S.H. largely contributed to the revision process. All the authors contributed substantial input to the final version of the manuscript.

## Declaration of interests

The authors declare no competing interests.

## STAR★Methods

### Key resources table

**Table:** 

REAGENT or RESOURCE	SOURCE	IDENTIFIER
**Antibodies**		

Anti- Digoxigenin-AP, Fab fragments	Roche	Cat#: 11093274910; RRID: AB_2734716
Anti-Digoxigenin-POD, Fab fragments	Roche	Cat#: 11207733910; RRID: AB_514500

**Chemicals, peptides, and recombinant proteins**		

sodium nifurstyrenate	UENO FOOD TECHNO INDUSTRY	Cat#: 210105
Tricainemethanesulfonate/ethyl3-aminobenzoate methanesulfonate	Sigma-Aldrich	Cat#: E10521
Inulin	Tokyo Chemical Industry	Cat#: I1067
Paraformaldehyde	Sigma-Aldrich	Cat#: 158127-3 KG
Paraplastplus	Leica Biosystems	Cat#: 39602004
MAS-GP-coated slide	Matsunami Glass	Cat#: 5079W
Kernechtrot Stain Solution	MUTO PURE CHEMICALS	Cat#: 40871
Permount	ThermoFisherScientific	Cat#: SP15-500
ISOGEN	Nippon gene	Cat#: 319-90211
Turbo DNase	Thermo Fisher Scientific	Cat#: AM2238
Calf Thymus DNA	Worthington Biochemical Corporation	Cat#: LS002105
4-Nitro blue tetrazolium chloride (NBT)	Sigma-Aldrich	Cat#: N6639-250 MG
5-Bromo-4-chloro-3-indolyl phosphate (BCIP)	Sigma-Aldrich	Cat#: B8503-100M
Proteinase K	Sigma-Aldrich	Cat#: SRE0005-15 ML

**Critical commercial assays**		

High-Capacity cDNA Reverse Transcription Kit	Thermo Fisher Scientific	Cat#: 4374967
TURBO DNase-free kit	Thermo Fisher Scientific	Cat#: AM1907
KAPA Taq EXtra	Roche	Cat#: KK3009
MIDORI Green Xtra	NIPPON Genetics	Cat#: MG10
Primestar GXL	Takara Bio	Cat#: R050A
Wizard SV Gel and PCR Clean-up System	Promega	Cat#: A2920
DIG RNA Labeling Kit (SP6/T7)	Roche	Cat#:11175025910
TSA Plus Biotin	Akoya Bioscience	Cat#: NEL749A001KT
ABC Elite Kit	Vector labratory	Cat#: PK-6100
Streptavidin Alexa Fluor 488 conjugate	Invitrogen	Cat#: S11223
Methyl Green Solution	Wako	Cat#: 138-12701
KAPA SYBR Fast qPCR kit	NIPPON Genetics	Cat#: KK4605

**Deposited data**		

Raw sequencing data for cloning and RNA probe synthesis	This study	GenBank: LC874643, LC874644, LC874645, LC874646, LC874647, LC874648
Squalomix Sequence Archive	Nishimura et al.^63^	Squalomix: https://transcriptome.riken.jp/squalomix/blast/
RNA-seq of stingray kidney	Aburatani et al.^23^	GenBank: DRX363233, DRX363234, DRX363235, DRX363236, DRX363237, DRX363238
See Tables S2 and S3	This study	N/A

**Experimental models: Organisms/strains**		

Red stingray (*Hemitrygon akajei*)	Wild caught	N/A

**Oligonucleotides**		

See Table S4	This study	N/A

**Recombinant DNA**		

pGEM-T Easy Vector	Promega	A1360

**Software and algorithms**		

MAFFT v7.505	Katoh and Standley^64^	https://mafft.cbrc.jp/alignment/software/
trimAl v1.4. rev15	Capella-Gutiérrez et al.^65^	https://vicfero.github.io/trimal/
RAxML v8.2.12	Hübner et al.^66^	https://cme.h-its.org/exelixis/web/software/raxml/
PhyloBayes v4.1c	Lartillot et al.^67^	https://bioweb.pasteur.fr/packages/pack@phylobayes@4.1c
ImageJ package Fiji	Schindelin et al.^68^	https://imagej.net/software/fiji/
Sequence Detection System software v2.4	Applied Biosystems	Cat# 4444202
Kyplot	Kyence	https://www.kyenslab.com/ja-jp/

**Other**		

polyethylene cannula SP-45	Natsume Seisakusyo	KN-392-1-SP45
polyethylene cannula SP-55	Natsume Seisakusyo	KN-392-1-SP55
atomic absorption spectrophotometer	Hitachi	Z5300
digital chloridometer	Jokoh	C-50AP
vapor pressure osmometer	Wescor	5600
DNA sequencer	Applied Biosystems	ABI PRISM 3100
digital camera	Nikon	DXM1200
confocal laser scanning microscope	Olympus	FV1000-D BX61WI
Nanodrop	Thermo Fisher Scientific	Cat#: 13-400-525

### Experimental model and study participant details

#### Animals

Male and female red stingrays (*Hemitrygon akajei*) (Muller and Henle, 1841), were caught at Kinkai Bay or Sagami Bay in 2022 and 2023 (average disc length = 52.8 ± 1.8 cm, average body mass = 4.7 ± 0.5 kg) and transported to the Atmosphere and Ocean Research Institute (AORI), The University of Tokyo. After treatment by sodium nifurstyrenate (UENO FOOD TECHNO INDUSTRY, Tokyo, Japan) up to 10 h, stingrays were kept in 500 L tanks filled with recirculated full-strength SW (35‰–36‰) under constant condition (20°C, 12L: 12D). They were fed with chopped squid or sardine *ad libitum*. All procedures for animal experiments were approved by the Animal Ethics Committee of Atmosphere and Ocean Research Institute of The University of Tokyo (P19-2). The present study was carried out in compliance with the ARRIVE guidelines. To avoid possible contamination with male seminal fluid during urine collection, only female fish were used. In all other experiments, both male and female fish were included in the analysis, and no sex-based differences were observed.

### Method details

#### Experiment 1: Repeated urine collection during transfer experiments

Stingrays were sequentially transferred from full-strength SW (SW group) to 5% SW (5% SW group) and then to full-strength SW (RSW group). Stingrays were unfed 2 days before the experiment and during whole the experiment. To lower the osmotic stress that may add to the surgical stress, the final dilution of environmental water was stopped at 5% SW instead of FW. Acclimation to 5% SW was conducted according to Aburatani et al.^23^ (see also Figure S8). In short, after urine collection in SW environment (days 1–3), 10–15% dilution of rearing SW was conducted daily using dechlorinated FW with interposing for 4 days around 50% SW (17‰–18‰) as acclimation period (days 7–10). The dilution of SW was stopped at 5% SW (<2‰) instead of FW to avoid excess stressful condition caused by the osmotic stress and the repeated surgical stress. After 3 days in 5% SW (days 14–16), during which urine collection was conducted, the stingrays were transferred back to full-strength SW (Figure S8). For the first and second days (days 17 and 18), full-strength SW was added to achieve a salinity of 30% SW and 60% SW, respectively. The stingrays were kept in 60% SW for 3 days for acclimation to high salinity (days 18–20), and salinity of the tanks was then elevated by 10–15% daily with artificial 200% SW until reaching 100% SW (35‰–36‰) on day 23.

Urine collection was conducted once for each in SW, 5% SW and RSW (Figure S9A). More than 6 h after reaching the target salinity, stingrays were anesthetized with 0.02% (w/v) ethyl 3-aminobenzoate methanesulfonate (MS-222; Sigma-Aldrich, MO, USA) buffered with equal concentration of sodium bicarbonate (Sigma-Aldrich). The blood sample for inulin blank was obtained from the caudal vessel using a 22G needle with a syringe. To examine GFR, 10% inulin (Tokyo Chemical Industry, Tokyo, Japan) was prepared by dissolving in elasmobranch Ringer (KCl; 6.6 mM, Na_2_HPO_4_; 1.4 mM, Na_2_SO_4_; 2.4 mM, TMAO; 56 mM, HEPES; 10 mM, NaCl; 255 mM, Urea; 424 mM, MgCl_2_.6H_2_O; 4 mM, CaCl_2_.2H_2_O; 6.6 mM, NaHCO_3_; 1.44 mM, pH = 7.55, for SW and RSW groups) or 60%-diluted elasmobranch Ringer (for 5% SW group), and were intramuscularly injected (2 mL/kg body mass) into the dorsal muscle. Inulin was administered first in SW, and then after the tank water reached a new target salinity (5% SW or RSW); thus, each individual received inulin injections multiple times depending on the number of acclimation steps completed, up to three times. A polyethylene cannula (SP-45 or 55 with handmade-balloon at 1 cm below the tip; Natsume Seisakusyo, Tokyo, Japan) was subsequently inserted into the urinary papilla and was secured by ligature to surrounding tissue with silk suture (Figure S9A). The other end of the cannula was connected to a 15- or 50-mL conical tube with airway and secured by epoxy resin (Figure S9B). Stingrays were recovered from anesthesia by irrigating the gills with aerated holding water and then returned to a 100 L container tank (700 cm × 500 cm × 410 cm) for urine collection (Figure S9B). Urine excreted from conscious stingrays was collected into the conical tube placed outside the experimental container. Urine collection was started more than 24 h after the inulin injection and recovery from anesthesia. The durations of urine sample collection times were typically about 2–5 h in SW and RSW individuals and about 0.5 h in 5% SW-acclimated individuals, depending on the urine flow rate. Immediately after the urine collection, the stingrays were anesthetized again to obtain blood samples as described above and to detach the urine collection device. Stingrays were then recovered from anesthesia, returned to a 500 L holding tank, and left for 1 or 2 nights in order to recover from the surgical stress. Five stingrays went through 3 consecutive acclimations (SW, 5% SW, and RSW). However, urine collection from 2 out of 5 individuals in RSW was unsuccessful due to damage of the urinary papilla after repeated surgeries. Therefore, we conducted the same experimental regime with 3 additional stingrays but skipped the urine collection in SW to preserve the integrity of urinary papilla during urine collection in 5% SW and RSW.

Blood samples were centrifuged at 10,000 g at 4°C for 10 min to obtain plasma. Plasma and urine samples were stored at −30°C until analysis. Sodium and chloride ion concentrations were determined using an atomic absorption spectrophotometer (Z5300; Hitachi, Tokyo, Japan) and a digital chloridometer (C-50AP; Jokoh, Japan), respectively. Osmolality was measured by a vapor pressure osmometer (5600; Wescor, UT, USA). Urea and inulin concentrations were colorimetrically determined according to Rahmatullah and Boyde^69^ and Roe et al.,^70^ respectively. The urine volume was gravimetrically measured, given the relative density as 1 g/mL. UFR and GFR were calculated as.

UFR (mL/kg/h) = Uv (mL) ÷ body wight (kg) ÷ collection time (hour), and

GFR (mL/kg/h) = UFR (mL/kg/h) × U_Inulin_ (mg/dL) ÷ P_Inulin_ (mg/dL). where Uv, U_Inulin,_ and P_Inulin_ represent urine volume, inulin concentration in urine, and inulin concentration in plasma, respectively. Based on the UFR and GFR, percentage of water reabsorption was calculated as.

Water reabsorption percentage (%) = {GFR (mL/kg/h) - UFR (mL/kg/h)} ÷ GFR (mL/kg/h) × 100.

We further calculated solute reabsorption rates and solute reabsorbed percentages as.

Solute reabsorption rates (μmol/kg/h) = P_solute_ (mM) × GFR (mL/kg/h) - U_solute_ (mM) × UFR (mL/kg/h), and.

Solute reabsorption percentages (%) = solute reabsorption rates (μmol/kg/h) ÷ GFR (mL/kg/h) ÷ P_solute_ (mM) × 100.

#### Experiment 2: Tissue collection from SW, FW and RSW groups

For molecular and histochemical analyses of aquaporins, frozen and fixed kidney samples of SW- and FW-acclimated red stingrays were used from the previous study (6 individuals each for SW and FW).^23^ In addition, kidney samples of RSW group were obtained. For this purpose, 9 stingrays (2 for SW group, 2 for FW group, and 5 for RSW group) were transported to AORI and kept at least for 2 days without feeding to acclimate to the experimental tank. No surgery was performed in Experiment 2. The FW and RSW transfer experiments were conducted as described above, excluding surgical procedure for urine collection. At the sampling points, stingrays were euthanized with the same anesthetic described above. The whole kidneys were dissected out and separated into two-halves along the midline. One-half was immediately frozen by liquid nitrogen and stored at −80°C until RNA extraction. The other half was fixed in modified Bouin’s solution (saturated picric acid: formalin = 3:1) for 2 days at 4°C and then preserved in 70% ethanol at 4°C until use. Gill, liver, stomach, intestine, rectum, rectal gland, and interrenal gland were also sampled for analyzing gene expression distribution in different tissues.

#### Molecular phylogenetic analysis

Protein sequences used for the phylogenetic analysis were collected from the NCBI databases and Squalomix Sequence Archive (https://transcriptome.riken.jp/squalomix/blast/, Nishimura et al.^63^), and their accession IDs are included in Tables S2 and S3. The amino acid sequences were deduced from gene model of red stingray genome sequence (BioProject ID GCF_048418815.1) and were aligned with the MAFFT v7.505^64^ using the L-INS-i method. The aligned sequences were trimmed with trimAl v1.4. rev15^65^ to remove unreliably aligned sites using the ‘-gappyout’ option. The maximum-likelihood tree was inferred with RAxML v8.2.12^66^ using the PROTCATWAG model, and for evaluating the confidence of the nodes, the rapid bootstrap resampling with 100 replicates was performed. Molecular phylogenetic tree employing the Bayesian framework was inferred with PhyloBayes v4.1c^67^ using the CAT-WAG-Γ model.

#### cDNA cloning and RNA probe synthesis

For the red stingray genes identified and phylogenetically annotated as above, TPM values of *Aqp*s expressed in the kidney were obtained from our previous RNA-seq data.^23^ Complementary DNA (cDNA) was synthesized using High-Capacity cDNA Reverse Transcription Kit (Thermo Fisher Scientific, MA, USA) from 2 μg of total RNA prepared by ISOGEN (Nippon Gene, Toyama, Japan) following by DNase (TURBO DNase-free kit, Thermo Fisher Scientific). Twelve kidney cDNA (SW = 6, FW = 6) were synthesized from the previously obtained RNA samples that were applied the same procedure with Experiment 2.^23^ Primer sets were designed to amplify target cDNA based on the contig sequence data from the transcriptome database (Table S4). PCR was performed using KAPA Taq Extra (Roche Applied Science, Mannheim, Germany, MA, USA) with the cDNA as templates (30 s at 95°C, 30s at 55°C, 1 min at 72°C, 27–40 cycles) after 3 min at 95°C. The amplicons were visualized by electrophoresis on 1.2% agarose gels containing a fluorescent dye (MIDORI Green Xtra, NIPPON Genetics, Tokyo, Japan). The amplicons were then ligated into pGEM-T Easy Vector (Promega, WI, USA) and sequenced by an automated DNA sequencer (ABI PRISM 3100, Applied Biosystems, CA, USA).

To synthesize a digoxigenin (DIG)-labeled RNA probe, the insert region of the sequenced plasmid was amplified with Primestar GXL (Takara Bio, Shiga, Japan) using the vector-specific M13 Forward and Reverse primers and subsequently purified with Wizard SV Gel and PCR Clean-up System (Promega). The purified DNA contains T7 and SP6 promotor sequences flanking the insert. Antisense and sense RNA probes were then synthesized from the purified DNA fragments using DIG RNA Labeling Kit (Roche) with either T7 or SP6 RNA polymerase according to the manufacturer’s protocols.

#### Histochemistry

The fixed kidney was embedded in Paraplast (Leica Biosystems, Wetzlar, Germany) and sectioned at 7 μm thickness. Kidney sections from SW control, FW-acclimated, and RSW-acclimated stingrays were mounted onto a single MAS-GP-coated slide (Matsunami Glass, Osaka, Japan) for comparison of the signal intensities among individuals of 3 groups with the same staining procedure. Deparaffinized sections were treated with 2.5 μg/mL proteinase K (Sigma-Aldrich) and then hybridized with DIG-labeled RNA probes in hybridization buffer (50% formamide, 5 × SSC, 40 μg/mL calf thymus DNA) at 58°C for 2 days. After hybridization, sections were serially washed in 2 × SSC for 30 min at room temperature, 2 × SSC for 1 h at 65°C, and 0.1 × SSC for 1 h at 65°C. The hybridized RNA probes were detected using Anti-Digoxigenin-AP, Fab fragment (1:5,000, Roche Applied Science). Hybridization signals were visualized with 4-nitro blue tetrazolium chloride (Sigma-Aldrich) and X-phosphate/5-bromo-4-chloro-3-indolyl-phosphate (Sigma-Aldrich). Sections were counterstained with Kernechtrot Stain Solution (MUTO PURE CHEMICALS, Tokyo, Japan), dehydrated with a graded series of ethanol, and mounted using Permount (Fisher Chemical, NJ, USA). For morphological observation, periodic acid and Schiff reagent (PAS) and subsequent hematoxylin staining was conducted. For PAS, Deparaffinized sections were oxidized in 0.5% periodic acid solution (Wako) for 5 min. After washing in tap water and distilled water, sections were placed in Schiff’s reagent (Wako) for 15 min, and then rinsed with wash solution (10% sulfurous acid:1N hydrochloric acid: distilled water = 24 mL:20 mL:400 mL) three times. Sections were then counterstained with Mayer’s hematoxylin (Wako). Stained sections were mounted with Permount (Thermo Fisher Scientific). Brightfield images were obtained using a digital camera (DXM1200; Nikon, Tokyo, Japan). Since the cells that express *Aqp1* mRNA were difficult to observe under bright field conditions, fluorescence *in situ* hybridization (FISH) was also conducted. After hybridization and washing with the same protocol, the hybridized probe was incubated with Anti-Digoxigenin-POD, Fab fragments (1:500, Roche Applied Science). The sections were washed with 1M Tris and 1.5M NaCl buffer (pH = 7.5) and subsequently incubated sequentially with TSA Plus Biotin (Akoya Bioscience, MA, USA), Abidin-Biotin-Complex (ABC Elite Kit; Vector Laboratory, CA, USA), and then Streptavidin Alexa Fluor 488 conjugate (Invitrogen, MA, USA). The nuclei were stained with Methyl Green Solution (Wako). Fluorescence images were obtained using a confocal laser scanning microscope (FV1000-D BX61WI; Olympus, Tokyo, Japan) and were processed by ImageJ package Fiji.^68^

#### Quantitative real-time PCR

The mRNA expression levels were measured by quantitative real-time PCR (qPCR) using 7900HT Fast Real-Time PCR System (Applied Biosystems). To obtain the best proxy of the absolute copy numbers, the concentrations of plasmids containing target sequences were measured by Nanodrop (Thermo Fisher Scientific), and subsequently diluted to 1,000,000 copies/μL, based on their molecular weight. These adjusted plasmids were used as standard templates for absolute quantification in qPCR assay. One μL of cDNA, serially diluted plasmids as the standards or water as negative controls were mixed with KAPA SYBR FAST qPCR Kit (Kapa Biosystems) and primers (Table S4, 0.01–0.04 μM in final concentration) in 10 μL of reaction volume (15 s at 95°C, 1 min at 60°C, 40 cycles after 2 min at 50°C and 10 min at 95°C). Following confirmation of the single peak of amplicons in a melt curve analysis, the copy numbers were calculated using Sequence Detection System software v2.4 (Applied Biosystems). The cDNAs and standards were duplicated and triplicated, respectively. Copy numbers of target genes were normalized by those of *Ef1α1* as an internal control. Primer sets for qPCR assay (Table S4) were designed using PrimerQuest (https://www.idtdna.com/Primerquest/Home/Index).

#### Transmission electron microscopy (TEM)

For transmission electron microscopy, a kidney of female stingray reared in SW (DL = 36.5 cm, BW = 1.4 kg) was dissected out under stereomicroscope and fixed with 2% paraformaldehyde and 2.5% glutaraldehyde in 0.1 M cacodylate buffer (CB; pH = 7.4) for 1 day. After washing in CB, the kidney was additionally fixed with 1% osmium tetroxide for 1 h. The sample was dehydrated with a series of ethanol and propylene oxide, and subsequently embedded in Epon 812. Ultrathin sections were cut using a diamond knife and were mounted on copper grids. These sections were stained with uranyl acetate and lead citrate and were observed using a transmission electron microscope (TEM, JEM-1400; JEOL, Tokyo, Japan).

### Quantification and statistical analysis

Statistical analysis was performed using Kyplot 6.0 software (Kyenslab, Tokyo, Japan). Values were expressed as means ± s.e.m. Each *n* number represents the number of animals used for the analysis. After the assumption of normality with Shapiro-Wilk test, data were compared using Tukey-Kramer multiple comparison test. When the values were not normally distributed, Steel-Dwass multiple comparison test was used. *p* < 0.05 was considered as statistically significant.

## References

[1] Grant M.I., Kyne P.M., Simpfendorfer C.A., White W.T., Chin A. (2019). Categorising use patterns of non-marine environments by elasmobranchs and a review of their extinction risk. Rev. Fish Biol. Fish..

[2] Yancey P.H. (2015).

[3] Pang P.K., Griffith R.W., Atz J.W. (1977). Osmoregulation in elasmobranchs. Am. Zool..

[4] Smith H.W. (1936). The retention and physiological role of urea in Elasmobranchii. Biol. Rev..

[5] Hazon N., Wells A., Pillans R.D., Good J.P., Gary Anderson W., Franklin C.E. (2003). Urea based osmoregulation and endocrine control in elasmobranch fish with special reference to euryhalinity. Comp. Biochem. Physiol. B.

[6] Ballantyne J.S., Robinson J.W. (2010). Freshwater elasmobranchs: a review of their physiology and biochemistry. J. Comp. Physiol. B.

[7] Edwards S.L., Marshall W.S. (2012).

[8] Kültz D. (2015). Physiological mechanisms used by fish to cope with salinity stress. J. Exp. Biol..

[9] Breves J.P., Shaughnessy C.A. (2024). Endocrine control of gill ionocyte function in euryhaline fishes. J. Comp. Physiol. B.

[10] Nordlie F.G. (2009). Environmental influences on regulation of blood plasma/serum components in teleost fishes: a review. Rev. Fish Biol. Fish..

[11] Varsamos S., Nebel C., Charmantier G. (2005). Ontogeny of osmoregulation in postembryonic fish: a review. Comp. Biochem. Physiol. A.

[12] Oide H., Utida S. (1968). Changes in intestinal absorption and renal excretion of water during adaptation to sea-water in the Japanese eel. Marine Biol..

[13] Elger E., Elger B., Hentschel H., Stolte H. (1987). Adaptation of renal function to hypotonic medium in the winter flounder (*Pseudopleuronectes americanus*). J. Comp. Physiol. B.

[14] Talbot C., Eddy F.B., Potts W.T.W., Primmett D.R.N. (1989). Renal function in migrating adult atlantic salmon, *Salmo salar* L. Comp. Biochem. Physiol. A.

[15] Janech M.G., Fitzgibbon W.R., Ploth D.W., Lacy E.R., Miller D.H. (2006). Effect of low environmental salinity on plasma composition and renal function of the Atlantic stingray, a euryhaline elasmobranch. Am. J. Physiol..

[16] Hunn J.B., Willford W.A. (1970). The effect of anesthetization and urinary bladder catheterization on renal function of rainbow trout. Comp. Biochem. Physiol..

[17] Carter K.M., Woodley C.M., Brown R.S. (2011). A review of tricaine methanesulfonate for anesthesia of fish. Rev. Fish Biol. Fish..

[18] Lacy E.R., Reale E. (1985). The elasmobranch kidney: II. Sequence and structure of the nephrons. Anat. Embryol..

[19] Hyodo S., Hoogenboom J.L., Anderson W.G., Alderman S.L., Gillis T.E. (2024). Encyclopedia of Fish Physiology.

[20] Hyodo S., Kakumura K., Takagi W., Hasegawa K., Yamaguchi Y. (2014). Morphological and functional characteristics of the kidney of cartilaginous fishes: with special reference to urea reabsorption. Am. J. Physiol..

[21] Chen L., Chou C.L., Knepper M.A. (2021). A comprehensive map of mRNAs and their isoforms across all 14 renal tubule segments of mouse. J. Am. Soc. Nephrol..

[22] Takvam M., Wood C.M., Kryvi H., Nilsen T.O. (2021). Ion transporters and osmoregulation in the kidney of teleost fishes as a function of salinity. Front. Physiol..

[23] Aburatani N., Takagi W., Wong M.K.S., Kadota M., Kuraku S., Tokunaga K., Kofuji K., Saito K., Godo W., Sakamoto T., Hyodo S. (2022). Molecular and morphological investigation on the renal mechanisms enabling euryhalinity of red stingray *Hemitrygon akajei*. Front. Physiol..

[24] Imaseki I., Wakabayashi M., Hara Y., Watanabe T., Takabe S., Kakumura K., Honda Y., Ueda K., Murakumo K., Matsumoto R. (2019). Comprehensive analysis of genes contributing to euryhalinity in the bull shark, *Carcharhinus leucas*; Na+-Cl− co-transporter is one of the key renal factors upregulated in acclimation to low-salinity environment. J. Exp. Biol..

[25] Login F.H., Nejsum L.N. (2023). Aquaporin water channels: roles beyond renal water handling. Nat. Rev. Nephrol..

[26] Yokota S.D., Benyajati S., Dantzler W.H. (1985). Comparative aspects of glomerular filtration in vertebrates. Ren. Physiol..

[27] Wilson J.L.L., Miranda C.A., Knepper M.A. (2013). Vasopressin and the regulation of aquaporin-2. Clin. Exp. Nephrol..

[28] Kato A., Perry S.F. (2024). The kidney for osmoregulation. In: Alderman S.L., Gillis T.E. editors. Encyclopedia of Fish Physiology.

[29] Sato Y., Nishida M. (2010). Teleost fish with specific genome duplication as unique models of vertebrate evolution. Environ. Biol. Fishes.

[30] Smith H.W. (1931). The absorption and excretion of water and salts by the elasmobranch fishes: I. Fresh water elasmobranchs. Am. J. Physiol..

[31] Wells A., Anderson W.G., Hazon N. (2002). Development of an in situ perfused kidney preparation for elasmobranch fish: action of arginine vasotocin. Am. J. Physiol. Regul. Integr. Comp. Physiol..

[32] Goldstein L., Forster R.P. (1971). Osmoregulation and urea metabolism in the little skate *Raja erinacea*. Am. J. Physiol..

[33] Madsen S.S., Engelund M.B., Cutler C.P. (2015). Water transport and functional dynamics of aquaporins in osmoregulatory organs of fishes. Biol. Bull..

[34] Kuraku S., Sato M., Yoshida K., Uno Y. (2024). Genomic reconsideration of fish non-monophyly: why cannot we simply call them all ‘fish. Ichthyol. Res..

[35] Friedman P.A., Hebert S.C. (1990). Diluting segment in kidney of dogfish shark. I. Localization and characterization of chloride absorption. Am. J. Physiol..

[36] Aburatani N., Takagi W., Wong M.K.S., Kadota M., Kuraku S., Tokunaga K., Kofuji K., Saito K., Godo W., Sakamoto T., Hyodo S. (2020). Facilitated NaCl uptake in the highly developed bundle of the nephron in Japanese red stingray *Hemitrygon akaje*i revealed by comparative anatomy and molecular mapping. Zool. Sci..

[37] Hebert S.C., Friedman P.A. (1990). Diluting segment in kidney of dogfish shark. II. Electrophysiology of apical membranes and cellular resistances. Am. J. Physiol..

[38] Watanabe S., Kaneko T., Aida K. (2005). Aquaporin-3 expressed in the basolateral membrane of gill chloride cells in Mozambique tilapia Oreochromis mossambicus adapted to freshwater and seawater. J. Exp. Biol..

[39] Cutler C.P., Martinez A.S., Cramb G. (2007). The role of aquaporin 3 in teleost fish. Comp. Biochem. Physiol. A.

[40] Tipsmark C.K., Sørensen K.J., Madsen S.S. (2010). Aquaporin expression dynamics in osmoregulatory tissues of Atlantic salmon during smoltification and seawater acclimation. J. Exp. Biol..

[41] Lacy E.R., Reale E. (1986). The elasmobranch kidney. III Fine structure of the peritubular sheath. Anat. Embryol..

[42] Boylan J.W. (1972). A model for passive urea reabsorption in the elasmobranch kidney. J. Comp. Physiol..

[43] Biemesderfer D., Payne J.A., Lytle C.Y., Forbush B. (1996). Immunocytochemical studies of the Na-K-Cl cotransporter of shark kidney. Am. J. Physiol..

[44] Kakumura K., Takabe S., Takagi W., Hasegawa K., Konno N., Bell J.D., Toop T., Donald J.A., Kaneko T., Hyodo S. (2015). Morphological and molecular investigations of the holocephalan elephant fish nephron: the existence of a countercurrent-like configuration and two separate diluting segments in the distal tubule. Cell Tissue Res..

[45] Hyodo S., Katoh F., Kaneko T., Takei Y. (2004). A facilitative urea transporter is localized in the renal collecting tubule of the dogfish *Triakis scyllia*. J. Exp. Biol..

[46] Cutler C.P., Harmon S., Walsh J., Burch K. (2012). Characterization of aquaporin 4 protein expression and localization in tissues of the dogfish (*Squalus acanthias*). Front. Physiol..

[47] Cutler C.P., Kurt K., Campbell K.E., Ojo T. (2022). Aquaporin (AQP) channels in the spiny dogfish, *Squalus acanthias* II: Localization of AQP3, AQP4 and AQP15 in the kidney. Comp. Biochem. Physiol. B.

[48] Robertson G.L., Shelton R.L., Athar S. (1976). The osmoregulation of vasopressin. Kidney Int..

[49] Bie P. (1980). Osmoreceptors, vasopressin, and control of renal water excretion. Physiol. Rev..

[50] Yamaguchi Y., Kaiya H., Konno N., Iwata E., Miyazato M., Uchiyama M., Bell J.D., Toop T., Donald J.A., Brenner S. (2012). The fifth neurohypophysial hormone receptor is structurally related to the V2-type receptor but functionally similar to V1-type receptors. Gen. Comp. Endocrinol..

[51] Matsuzaki T., Yaguchi T., Shimizu K., Kita A., Ishibashi K., Takata K. (2017). The distribution and function of aquaporins in the kidney: resolved and unresolved questions. Anat. Sci. Int..

[52] Ellis L.V., Bollinger R.J., Weber H.M., Madsen S.S., Tipsmark C.K. (2019). Differential expression and localization of branchial AQP1 and AQP3 in Japanese medaka (*Oryzias latipes*). Cells.

[53] Ruhr I.M., Wood C.M., Schauer K.L., Wang Y., Mager E.M., Stanton B., Grosell M. (2020). Is aquaporin-3 involved in water-permeability changes in the killifish during hypoxia and normoxic recovery, in freshwater or seawater?. J. Exp. Zool..

[54] Lin Y.T., Wu S.Y., Lee T.H. (2023). Salinity effects on expression and localization of aquaporin 3 in gills of the euryhaline milkfish (*Chanos chanos*). J. Exp. Zool..

[55] Morgan R.L., Ballantyne J.S., Wright P.A. (2003). Regulation of a renal urea transporter with reduced salinity in a marine elasmobranch. J. Exp. Biol..

[56] Kyne P.M., Lucifora L.O. (2022). Carrier J.C., Simpfendorfer C.A., Heithaus M.R., Yopak K.E. editors. Biology of sharks and their relatives.

[57] Ballantyne J.S., Fraser D.I. (2012).

[58] Zigman S., Munro J., Lerman S. (1965). Effect of urea on the cold precipitation of protein in the lens of the dogfish. Nature.

[59] Yancey P.H., Somero G.N. (1980). Methylamine osmoregulatory solutes of elasmobranch fishes counteract urea inhibition of enzymes. J. Exp. Zool..

[60] Kanoh S., Noma T., Ito H., Tsureyama M., Funabara D. (2023). Myosin light chain of shark fast skeletal muscle exhibits intrinsic urea-resistibility. Sci. Rep..

[61] Windhager E.E., Gottschalk C.W., Berliner R.W., Giebisch G.H. (1987). Renal Physiology: People and Ideas.

[62] Chauvigné F., Ferré A., Cerdà J. (2021). The Xenopus oocyte as an expression system for functional analyses of fish aquaporins. Methods Mol. Biol..

[63] Nishimura O., Rozewicki J., Yamaguchi K., Tatsumi K., Ohishi Y., Ohta T., Yagura M., Niwa T., Tanegashima C., Teramura A. (2022). Squalomix: shark and ray genome analysis consortium and its data sharing platform. F1000Res..

[64] Katoh K., Standley D.M. (2013). MAFFT multiple sequence alignment software version 7: improvements in performance and usability. Mol. Biol. Evol..

[65] Capella-Gutiérrez S., Silla-Martínez J.M., Gabaldón T. (2009). trimAl: a tool for automated alignment trimming in large-scale phylogenetic analyses. Bioinformatics.

[66] Hübner L., Kozlov A.M., Hespe D., Sanders P., Stamatakis A. (2021). Exploring parallel MPI fault tolerance mechanisms for phylogenetic inference with RAxML-NG. Bioinformatics.

[67] Lartillot N., Rodrigue N., Stubbs D., Richer J. (2013). PhyloBayes MPI: phylogenetic reconstruction with infinite mixtures of profiles in a parallel environment. Syst. Biol..

[68] Schindelin J., Arganda-Carreras I., Frise E., Kaynig V., Longair M., Pietzsch T., Preibisch S., Rueden C., Saalfeld S., Schmid B. (2012). Fiji: an open-source platform for biological-image analysis. Nat. Methods.

[69] Rahmatullah M., Boyde T.R. (1980). Improvements in the determination of urea using diacetyl monoxime; methods with and without deproteinisation. Clin. Chim. Acta.

[70] Roe J.H., Epstein J.H., Goldstein N.P. (1949). A photometric method for the determination of inulin in plasma and urine. J. Biol. Chem..

