# Supplementary material for: Extensive urine production in euryhaline red stingray for adaptation to hypoosmotic environments
Source: iScience. 2025 Aug 20;28(9):113274. doi: 10.1016/j.isci.2025.113274 (PMC12496175; doi:10.1016/j.isci.2025.113274)
Supplement: Document S1. Figures S1–S9 and Tables S1–S4 [file mmc1.pdf]

iScience, Volume *28*

Supplemental information

Extensive urine production in euryhaline red stingray for adaptation to hypoosmotic environments

Naotaka Aburatani, Wataru Takagi, Marty Kwok-Shing Wong, Nobuhiro Ogawa, Shigehiro Kuraku, Mana Sato, Kazuhiro Saito, Waichiro Godo, Tatsuya Sakamoto, and Susumu Hyodo

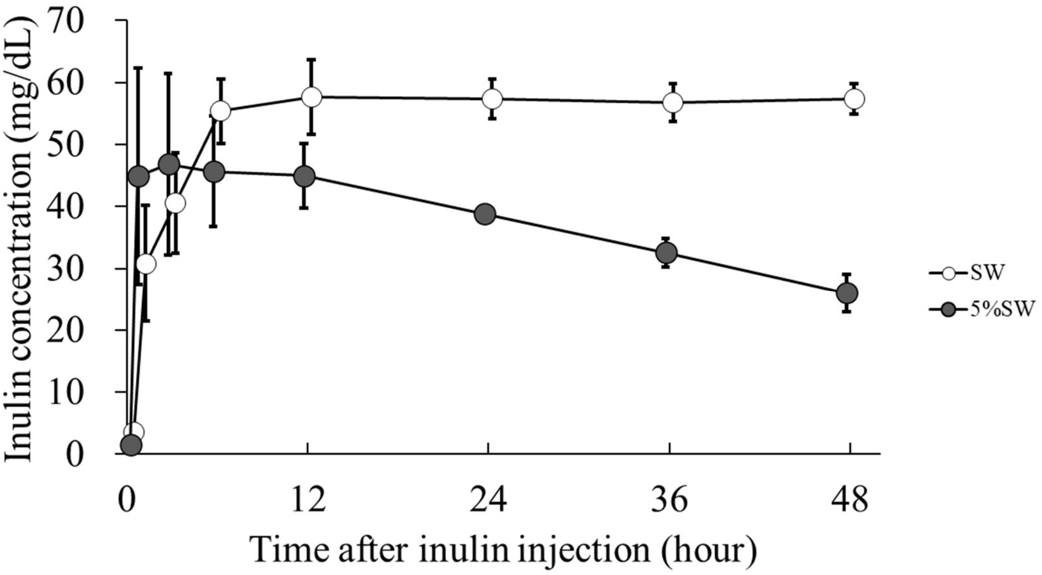

Fig. S1. The dynamics of plasma inulin concentration after administration. Open and filled markers indicate SW- and 5% SW-acclimated individuals, respectively (n = 3; values were expressed as average ± s.e.m.). Note that the plasma inulin level of SW group is stable between 24-48 hours after injection of inulin and thus inulin concentration of that regime can represent average inulin concentration during experimental periods.

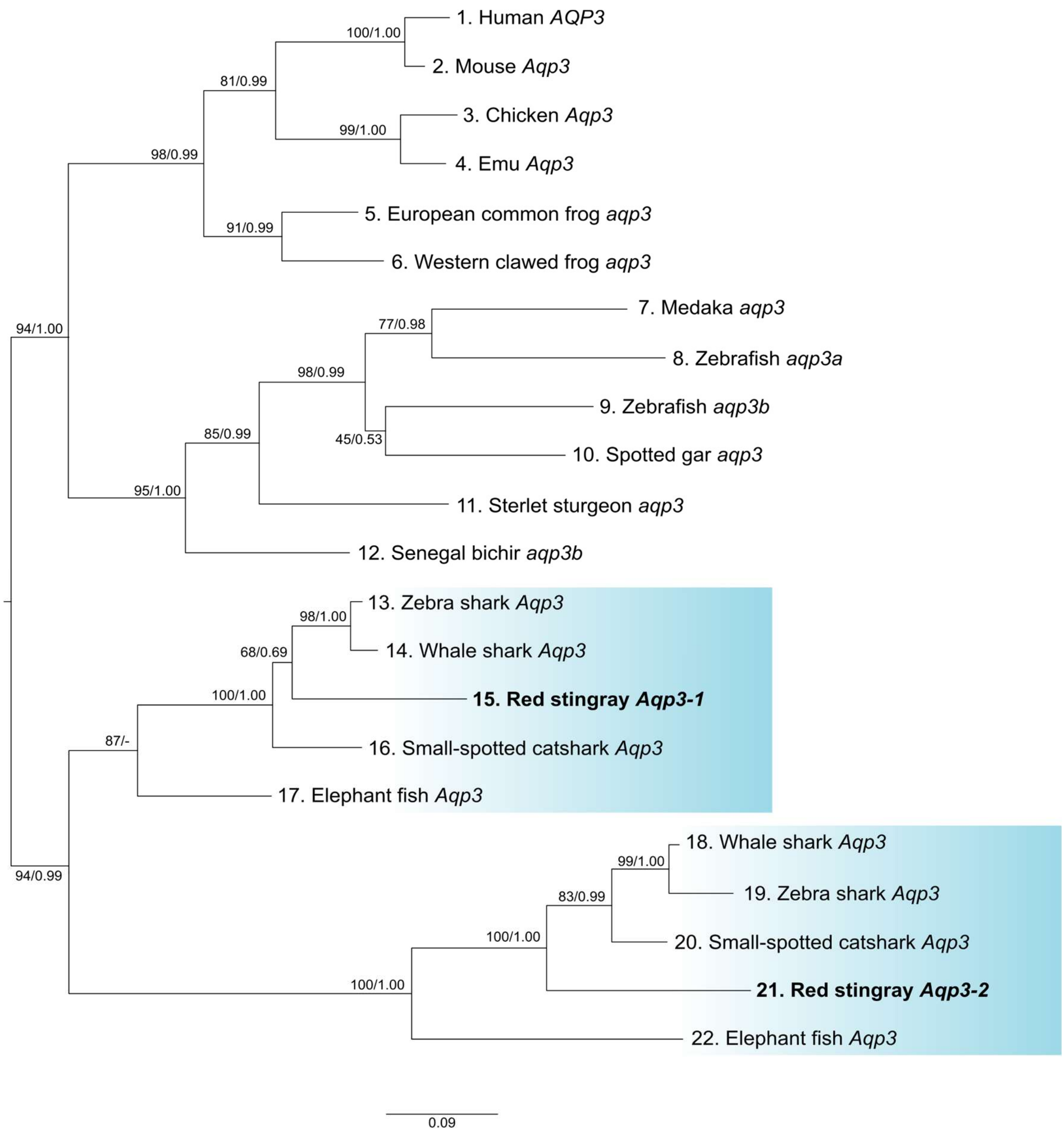

Fig. S2. Molecular phylogeny of the red stingray *Aqp3* genes. Their phylogenetic relationships in the aquaporin-3 subfamily inferred with the maximum-likelihood method with 266 residues in the amino acid sequence alignment (see Materials and Methods). The support values at nodes are bootstrap probabilities in the ML tree and posterior probabilities in the Bayesian inference in order (see Materials and Methods).

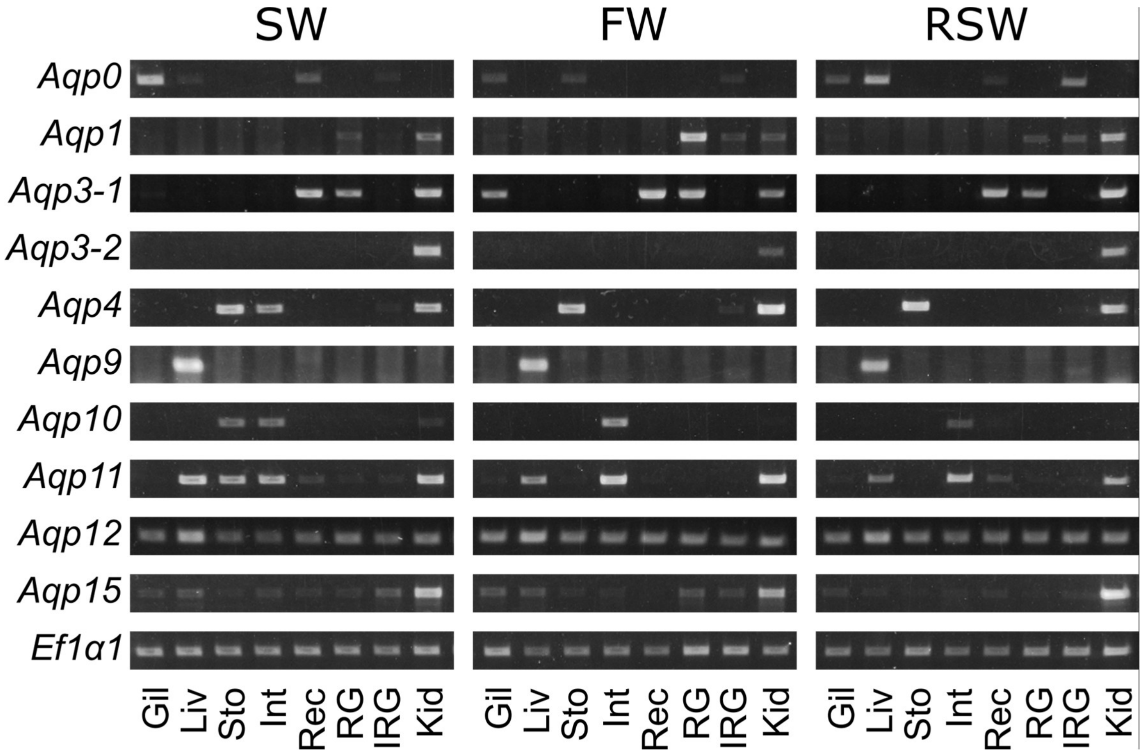

Fig. S3. The distribution of *Aqp*s in different tissues. The presence or absence of specific *Aqp* transcripts were tested by PCR and following electrophoresis. Gil, gill; Liv, liver; Sto, stomach; Int, intestine; Rec, rectum; RG, rectal gland; IRG, interrenal gland; Kid, kidney.

Table S1. The expression levels of *Aqp* s obtained by RNAseq of stingray kidey

transcript contig I	Annotation	Sequence ID	gene symbol	TPM value					
				SW1	SW2	SW3	FW1	FW2	FW3
DN50466_c10_g	aquaporin FA-CHIP-like [*Hypanus sabinus*]	XP_05982771	*Aqp1*	13.7	34.6	40.6	6.3	18.3	87.9
DN48015_c0_g1	aquaporin-3-like [*Hypanus sabinus*]	XP_05984558	*Aqp3-1*	19.1	39.2	25.9	6.9	4.6	2.0
DN53448_c13_g	aquaporin-3-like isoform X2 [Hypanus sabinus]	XP_05984559	*Aqp3-2*	57.3	22.0	67.7	1.5	2.4	2.0
DN53897_c2_g1	aquaporin-4 isoform X1 [*Hypanus sabinus*]	XP_05983330	*Aqp4*	2.5	1.3	1.7	5.6	11.1	6.6
DN135542_c0_g	aquaporin-9-like [*Hypanus sabinus*]	XP_05980854	*Aqp9*	0	0.2	0.2	0.4	0	0
DN56628_c8_g5	aquaporin-10-like [*Hypanus sabinus*]	XP_05983713	*Aqp10*	0.6	2.0	0.4	0.2	0	0.6
DN53121_c0_g1	aquaporin-11 [*Hypanus sabinus*]	XP_05981971	*Aqp11*	5.7	7.4	5.0	18.1	13.7	21.6
DN56250_c1_g1	aquaporin-5-like isoform X1 [*Hypanus sabinus*]	XP_05981178	*Aqp15*	26.8	21.5	78.2	3.5	8.2	2.7

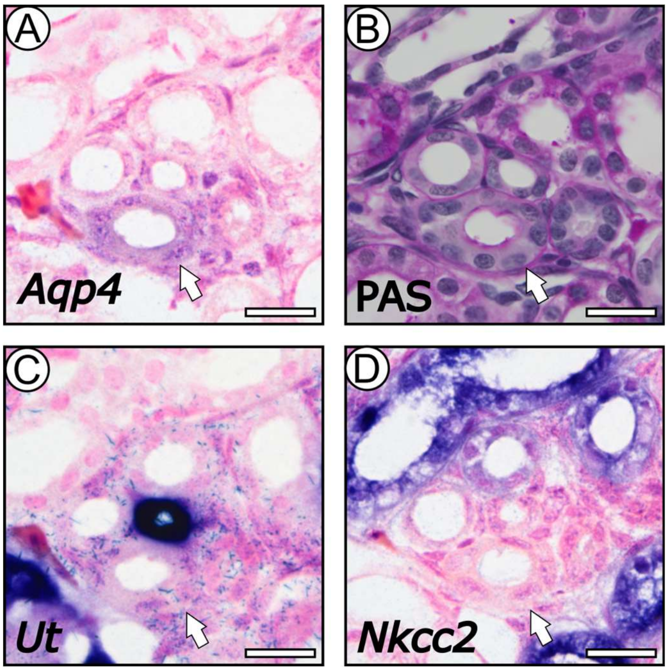

Fig. S4. Analysis of the nephron segment expressing *Aqp4* mRNA in the SW control individual by *in situ* hybridization and PAS-hematoxylin-staining against serial sections. Open arrows indicate *Aqp4*-positive segment in the bundle zone (**A**). The segment was apically PAS-positive (**B**, a marker for ascending PI), but negative for both *Ut* (**C**, a marker for CT), and *Nkcc2* (**D**, a marker for EDT), indicating *Aqp4* is expressed in the PI segment of the bundle zone. Bars, 20µm.

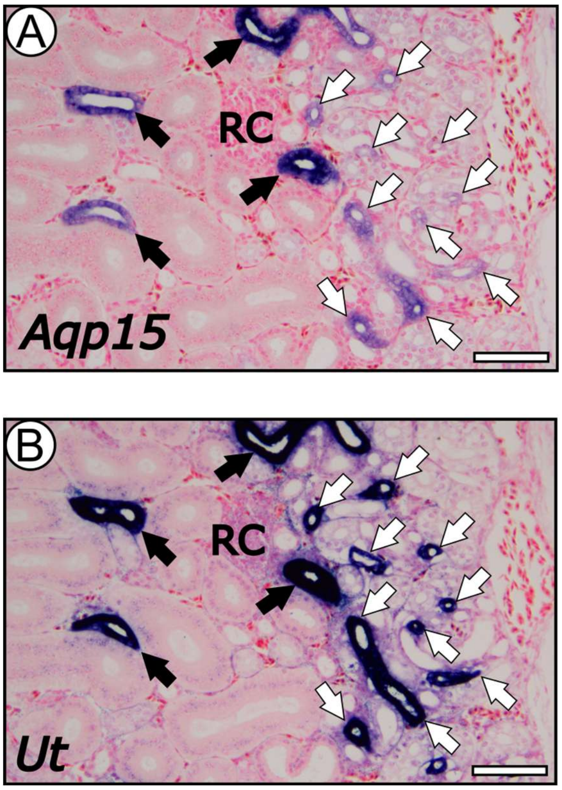

Fig. S5. Analysis of the nephron segment expressing *Aqp15* mRNA in the SW control individual. Filled and open arrows indicate the transitional portions of LDT in the sinus zone and the collecting tubules in bundle zone, respectively. The mRNA signals of both *Ut* (a marker) and *Aqp15* were colocalized in these segments. Bars, 100 μm.

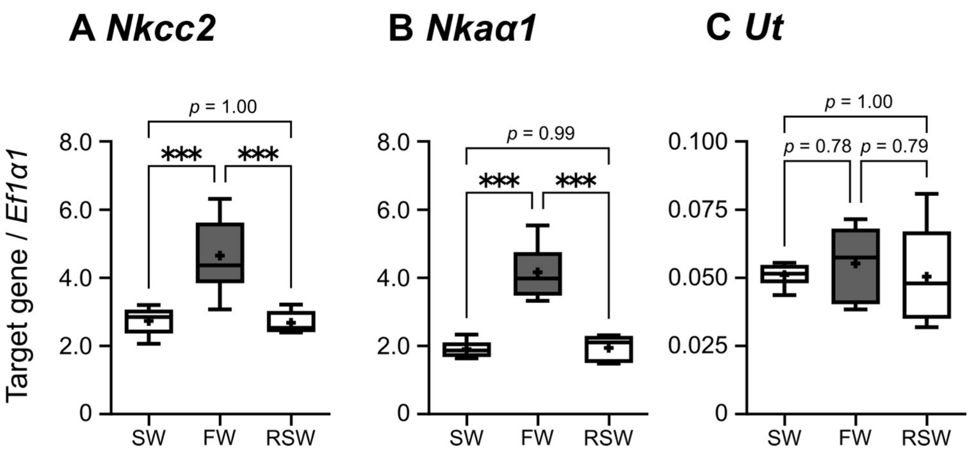

Fig. S6. Expression of transporter mRNAs in the kidney measured by qPCR. mRNAs for *Nkcc2* **(A)**, *Nkaα1* **(B)** and *Ut* **(C)** were quantified and normalized against those of elongation factor 1 alpha subunit 1 (*Ef1α1*). Statistically-significant difference among groups is shown with asterisks (*** P < 0.001, ***P* < 0.01, *n* = 8, 8, and 5 for SW, FW, and RSW groups, respectively).

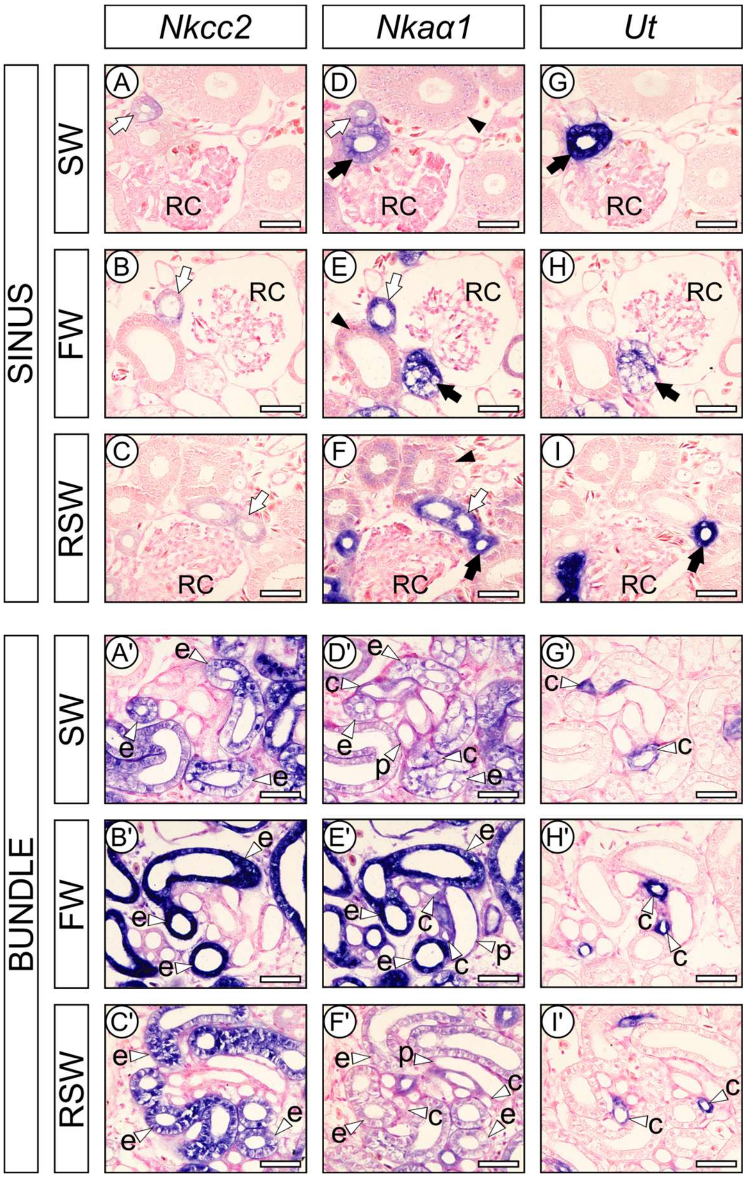

Fig. S7. *In situ* hybridization analysis of major transporter genes in the red stingray nephron. The respective mRNA signals in sinus zone (**A-I**) and bundle zone (**A'-I'**) of *Nkcc2* (**A-C** and **A'-C'**), *Nkaα1* (**D-F** and **D'-F'**) and *Ut* (**G-I** and **G'-I'**) are shown. Filled arrowheads indicate the PII (**D-F**). Open and filled arrows indicate the anterior and posterior LDT, respectively (**A-I**). RC, renal corpuscle. Open arrowheads indicate the PI (labeled with "p"), EDT (labeled with "e"), and CT (labeled with "c") in the bundle zone (**A'-I'**). Bars, 50 µm. Note that 1) the *Nkcc2* mRNA was intensely expressed in the EDT of FW-acclimated stingrays, but no difference was observed in LDT among different salinities, 2) concomitantly, prominent *Nkaα1* mRNA signals were observed in the EDT of FW-acclimated stingrays, and 3) the *Ut* signals in FW-acclimated group of CT was stronger, while those of posterior LDT was slightly weaker than other groups.

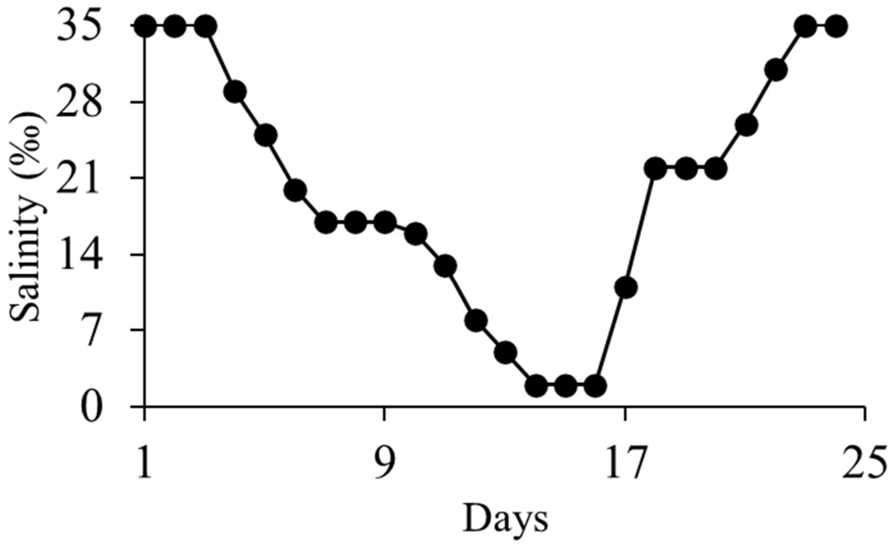

Fig. S8. Typical example of the salinity profile that stingrays experienced. Salinity of rearing water was gradually diluted and elevated by adding dechlorinated FW and then artificial SW, respectively. In this example, the stingray was kept in either full strength SW or 5% SW during day 1-3, 14-16, 23-24 to collect urine. Transfer to target salinities was interposed 3 or 4 days at day 7-10 and day 18-20 in the middle strength salinities (50-60% SW) for accommodation to osmotic stress.

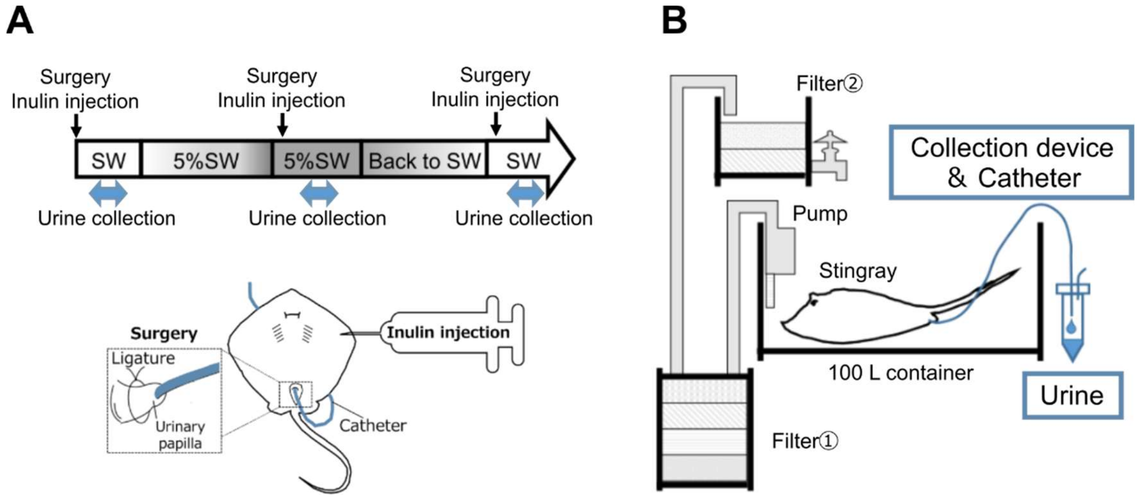

Fig. S9. Schematic drawing of repetitive surgery, inulin injection, and the setup for urine collection. **(A)** Anesthetized stingray was subject to injection of 10% inulin and attachment of the urine collecting device at each target salinity. **(B)** Urine is excreted from the catheterized urinary papilla of stingray into a conical tube setting outside of holding tank.

Table S2. Accession IDs used in this study

Orthogroup*	species	Accession ID
AQP8	*Homo sapiens*	NP_001160.2
AQP8	*Danio rerio*	ACV60540.2
AQP8	*Danio rerio*	NP_001073651.1
AQP8	*Danio rerio*	NP_001004661.1
AQP8	*Strongylocentrotus purpuratus*	XP_030854916.1
AQP8	*Patiria miniata*	XP_038075258.1
AQP8	*Patiria miniata*	XP_038069095.1
AQP8	*Strongylocentrotus purpuratus*	XP_030840396.1
AQP14	*Danio rerio*	XP_005174182.1
AQP14	*Hemitrygon akajei*	This study
AQP4	*Homo sapiens*	XP_011524244.1
AQP4	*Hemitrygon akajei*	This study
AQP4	*Danio rerio*	XP_009292895.1
cAQP	*Strongylocentrotus purpuratus*	XP_001190612.2
cAQP	*Patiria miniata*	XP_038051349.1
cAQP	*Patiria miniata*	XP_038078625.1
cAQP	*Strongylocentrotus purpuratus*	XP_001185961.1
AQP15	*Danio rerio*	XP_021327889.1
AQP15	*Hemitrygon akajei*	This study
AQP1	*Homo sapiens*	NP_932766.1
AQP1	*Danio rerio*	NP_996942.1
AQP1	*Danio rerio*	XP_021336241.1
AQP1	*Hemitrygon akajei*	This study
AQP5	*Homo sapiens*	NP_001642.1
AQP6	*Homo sapiens*	NP_001643.2
AQP2	*Homo sapiens*	NP_000477.1
AQP0	*Homo sapiens*	NP_036196.1
AQP0	*Hemitrygon akajei*	This study
AQP0	*Danio rerio*	NP_001003534.1
AQP0	*Danio rerio*	NP_001018356.1
AQP9	*Homo sapiens*	NP_066190.2
AQP9	*Hemitrygon akajei*	This study
AQP9	*Danio rerio*	NP_001171215.1
AQP9	*Danio rerio*	NP_001028268.1
AQP10	*Homo sapiens*	NP_536354.2
AQP10	*Danio rerio*	NP_001002349.1
AQP10	*Danio rerio*	ACB10577.1
AQP10	*Hemitrygon akajei*	This study
AQP3	*Homo sapiens*	NP_004916.1
AQP3	*Danio rerio*	NP_001159593.1
AQP3	*Danio rerio*	NP_998633.1
AQP3	*Hemitrygon akajei*	This study
AQP3	*Hemitrygon akajei*	This study
AQP7	*Homo sapiens*	NP_001161.1_
AQP7	*Danio rerio*	NP_956204.2
glpAQP	*Patiria miniata*	XP_038071748.1
glpAQP	*Patiria miniata*	XP_038073806.1
glpAQP	*Patiria miniata*	XP_038073167.1
glpAQP	*Strongylocentrotus purpuratus*	XP_792142.4
glpAQP	*Strongylocentrotus purpuratus*	XP_030833261.1
AQP11	*Homo sapiens*	NP_766627.1
AQP11	*Danio rerio*	AAH95775.1
AQP11	*Hemitrygon akajei*	This study
AQP12	*Homo sapiens*	NP_001095937.1
AQP12	*Homo sapiens*	NP_945349.1
AQP12	*Hemitrygon akajei*	This study
AQP12	*Danio rerio*	AAI21753.1
AQP11	*Strongylocentrotus purpuratus*	XP_003725070.1
AQP11	*Patiria miniata*	XP_038044509.1

Table S3. Accession IDs of AQP3 used in this study

Orthogroup*	species	Accession ID
AQP3	*Homo sapiens*	NP_004916.1
AQP3	*Mus musculus*	NP_057898.2
AQP3	*Gallus gallus*	XP_046792174.1
AQP3	*Dromaius novaehollandiae*	XP_025954210.1
AQP3	*Rana temporaria*	XP_040191985.1
AQP3	*Xenopus tropicalis*	XP_012818392.1
AQP3	*Oryzias latipes*	XP_004072505.1
AQP3	*Danio rerio*	NP_998633.1
AQP3	*Danio rerio*	NP_001159593.1
AQP3	*Lepisosteus oculatus*	XP_006626763.1
AQP3	*Acipenser ruthenus*	XP_033862580.1
AQP3	*Polypterus senegalus*	XP_039615087.1
AQP3	*Stegostoma tigrinum*	XP_048400543.1
AQP3	*Rhincodon typus*	XP_020377609.1
AQP3	*Hemitrygon akajei*	This study
AQP3	*Scyliorhinus canicula*	XP_038646608.1
AQP3	*Callorhinchus milii*	XP_007895329.1
AQP3	*Rhincodon typus*	XP_048454233.1
AQP3	*Stegostoma tigrinum*	XP_048401864.1
AQP3	*Scyliorhinus canicula*	XP_038646612.1
AQP3	*Hemitrygon akajei*	This study
AQP3	*Callorhinchus milii*	XP_007895330.1

Table S4. Primer sets used in the present study

Gene Name	Application		Primer sequence (5' to 3')
Aqp0	RT-PCR	Sense	AACGTGCTGCTGGTTTCTCT
		Antisense	CACTCCGAGGTTCCTGTTGT
Aqp1	RT-PCR / *in situ* hybridization	Sense	CCATCTTCGTCTTCCTCAGC
		Antisense	TCTCCATCCTCGCTTTGTCT
	qPCR	Sense	CCGCCATTCTCTACGACTTC
		Antisense	CACTTCATACTCCTCGCTCATAC
Aqp3-1	RT-PCR	Sense	TATTTTTGCCCCACTCCTTG
		Antisense	CGATGATCTGCGAACATGAA
	in situ hybridization	Sense	AATCTGTTGGGAAGGCAATG
		Antisense	GCTTCAGCATTCCAAGAAGG
	qPCR	Sense	GGACCGCTGCTCTTATTGT
		Antisense	CACCGTGAATCCAACTGTAAATG
Aqp3-2	RT-PCR / *in situ* hybridization	Sense	AGTTCACTTCCTGGCTCGAA
		Antisense	GTGAACAAGCGAGGTCCAAT
	qPCR	Sense	CAAATTGCTGTCCAGGAACAAA
		Antisense	GTGGAGCCACAACTTCTCTATAA
Aqp4	RT-PCR	Sense	AATCCTGCACGATCATTTGG
		Antisense	GAGGACATGCAAACTCCACA
	in situ hybridization	Sense	CGCCAGATTCTTCCTCACTC
		Antisense	TCCTGCTTTCCAAAACATCC
	qPCR	Sense	TACGAGGATCTGGCCATTAAAC
		Antisense	CTGCTTTCTTGTGCCCTTTG
Aqp9	RT-PCR	Sense	GGTCTTTCAATGGGCTTCAA
		Antisense	GCCATTTGGCTCTTTTGTGT
Aqp10	RT-PCR	Sense	GGTCACCACCTACCACAACC
		Antisense	TCGACTTCCTTCGACTTCGT
Aqp11	RT-PCR / *in situ* hybridization	Sense	CACAGTGGTGGTGTGTCAGG
		Antisense	TTGTTTCTCTGCATGCTGCT
	qPCR	Sense	CAGTCTGCTGACCCTTTCTAAG
		Antisense	TTGGCGTTGGGAGTGATG
Aqp12	RT-PCR	Sense	CCACGAGTACACGTTGGATG
		Antisense	AGCTGCTCCCTCAAACTTCA
Aqp15	RT-PCR / *in situ* hybridization	Sense	AGCAGCTAAAGCAGGAGGTG
		Antisense	AGAGGTCCCACCCAGAAGAT
	qPCR	Sense	CTAAAGCAGGAGGTGCACAG
		Antisense	CCGCAGAACACCAGCAC
Nkccc2	*in situ* hybridization	Sense	GCTGTAGCTGTGGCCATGTA
		Antisense	TTTTTCAGGCACTCTGCAAG
	qPCR	Sense	TATGAGCATGGTGTCTGGATTT
		Antisense	TTGGGTGCACTGACTAAAGAG
Nkaα1	*in situ* hybridization	Sense	AACGTATGGCACGGAAGAAC
		Antisense	TTGCAATAGCTTTGGCAGTG
	qPCR	Sense	GCTTCGTTGGACTGATGTCTAT
		Antisense	GTGATTGGATGGTCACCTGTTA
Ut	*in situ* hybridization	Sense	TGGCGATATGAAAGAATTTGG
		Antisense	GACGTACCCGGAGAAGAACA
	qPCR	Sense	TAGGGCAGAACATTGACATGAG
		Antisense	GGCATATTGGTCGGTCTCTTAC
Ef1α1	RT-PCR	Sense	AACGTGAACGTGGCATTACC
		Antisense	TTTAAGATGATTACTTGCGAAATGA
	qPCR	Sense	CCCAGAAACTGTGGCATTTG
		Antisense	GGTGATCTTCCAGCCTTTGA
